# Management of Endometriosis in the Workplaces: Preliminary Results from a Cross-Sectional Knowledge, Attitudes and Practices Study (Italy, 2026)

**DOI:** 10.3390/healthcare14152299

**Published:** 2026-07-29

**Authors:** Matteo Riccò, Marco Bottazzoli, Sara Scilli, Nicola Magnavita

**Affiliations:** 1 Servizio di Prevenzione e Sicurezza Negli Ambienti di Lavoro (SPSAL), AUSL-IRCCS di Reggio Emilia, Via Amendola n.2, 42122 Reggio Emilia, Italy; 2 Department of Otorhinolaryngology, Azienda Sanitaria Universitaria Integrata (ASUIT) Trento, 38122 Trento, Italy; marco.bottazzoli@asuit.tn.it; 3 Department of Biomedical, Metabolic and Neural Sciences, University of Modena and Reggio-Emilia, 41125 Modena, Italy; sara.scilli@gmail.com; 4 Department of Safety and Bioethics, Section of Occupational Health, Università Cattolica del Sacro Cuore, 00168 Rome, Italy; nicolamagnavita@gmail.com

**Keywords:** endometriosis, occupational medicine, occupational physicians, knowledge attitudes and practices, workplace accommodations, fitness for work, women’s health, occupational health

## Abstract

**Background:** Endometriosis is a chronic condition affecting a substantial proportion of women of reproductive age and leading to impaired quality of life. Despite its potential occupational implications, evidence on how occupational physicians (OPs) manage workers affected by endometriosis remains scarce. This study aimed to assess the knowledge, attitudes, and practices (KAP) regarding endometriosis among Italian OPs. **Methods**: A cross-sectional web-based survey was conducted between 1 and 18 April 2026 by recruiting a convenience sample of 155 occupational physicians. The questionnaire was specifically designed and investigated knowledge status, risk perception, perceived barriers to workplace management, and previous experience of participating professionals. **Results**: Most respondents considered themselves adequately informed about endometriosis (78.1%) and reported previous professional experience with affected workers (81.3%). Knowledge gaps were identified, particularly regarding disease prevalence (26.5% correct responses), familial predisposition (34.8%), and diagnostic pathways (31.6%). Overall, 65.8% of participants reported having previously issued conditioned fitness-for-work appraisals, most frequently recommending increased work breaks, avoidance of night shifts, or limitations on shift work. In multivariable analysis, male gender (adjusted odds ratio [aOR] 5.69, 95% confidence interval [95% CI] 1.84–17.58), participation in continuing medical education activities (aOR 85.72, 95% CI 6.60–1114.21), information obtained from colleagues (aOR 4.67, 95% CI 1.23–17.77), and employment in healthcare settings (aOR 5.91, 95% CI 1.92–18.16) were independently associated with having previously issued restricted fitness-for-work judgments, whereas relying exclusively on undergraduate medical education was negatively associated with this outcome (aOR 0.07, 95% CI 0.01–0.39). **Conclusions**: Among the sampled Italian OPs, endometriosis was recognized as a clinically relevant condition frequently encountered in daily practice. Continuous professional education, interdisciplinary exchange, and practical experience were more strongly associated with occupational decision-making than undergraduate training alone. These findings support the development of targeted educational interventions and shared occupational recommendations aimed at improving workplace management and promoting the inclusion and retention of women affected by endometriosis in the workforce.

## 1. Introduction

Endometriosis is a chronic inflammatory and estrogen-dependent condition defined by the presence of endometrial-like epithelial and/or stroma cells outside the endometrium and myometrium [1]. Extra-uterine endometrial-like tissue shows secretory activity and reacts to the hormonal changes occurring during the menstrual cycle, leading to inflammatory processes, locally referred to as endometriosis lesions [2,3], which cause substantial systemic clinical manifestations [1,4], ultimately resulting in increased healthcare utilization.

Approximately 7% to 15% of women of reproductive age are affected by endometriosis [1,4,5], with even higher proportions reported in specific subgroups, and is one of the leading causes of chronic pelvic pain, dysmenorrhea and/or dyspareunia, and infertility among women of reproductive age [1,2,5,6,7,8]. Given its symptoms and potential complications, endometriosis not only affects patients’ physical health but also has a substantial negative impact on psychological well-being [9,10], encompassing elevated rates of anxiety, depression, and psychological distress, all of which contribute to limitations in social functioning by impairing educational attainment, achievement of life goals, career development, and professional advancement [3,9,11]. Consequently, the impact of endometriosis on quality of life is considerable, particularly during the reproductive years [12].

Over recent decades, substantial advances have been made in the understanding of the epidemiology of endometriosis in the general population [1,2,4,5,6], the understanding of its socioeconomic impact remains largely uncertain [13] due to the limited availability of high-quality longitudinal studies [13,14]. For example, in a recent study on 34,490 patients from Denmark, women with endometriosis lost on average 0.26 years of working life (95% CI 0.17 to 0.37) compared to the general female population, mainly because of sick leave and disability pension [15]. In addition to absenteeism, endometriosis is associated with substantial presenteeism, whereby affected women remain at work despite symptoms, often experiencing marked reductions in productivity [1,14,16,17]. On the other hand, endometriosis, particularly when associated with severe symptoms, usually emerges and worsens during adolescence and early adulthood, thereby affecting critical periods of personal, educational, and occupational development [14]. Not coincidentally, women with surgically confirmed endometriosis have been reported to be employed in less demanding or part-time occupations (Odds Ratio [OR] 1.26, 95% CI 0.94 to 1.68), and to be unemployed (OR 1.85, 95% CI 1.16 to 2.96) compared with their occupational status before diagnosis [18]. Moreover, women affected by endometriosis are less likely to achieve higher levels of education and more likely to occupy lower-level occupational positions [13,18,19], possibly because of the long-term consequences of disabling symptoms and chronic pain [14].

Occupational implications have only recently been acknowledged as important public health issues and remain poorly investigated [12,13,14,15,18,19,20]. While several international scientific societies have developed recommendations regarding its diagnosis and clinical management, specific guidance addressing workplace accommodations, fitness-for-work evaluations, and occupational management strategies remains scarce [16,21,22,23], and the management of affected workers still relies on a limited evidence base.

In countries where occupational physicians (OPs), the medical professionals responsible for occupational health surveillance and health promotion in the workplace [24,25], are implemented by the local Occupational Health and Safety Legislation (e.g., Italy, France, Belgium, and Germany), they are routinely involved in workplace adaptation processes and in advising both employers and workers regarding the benefits and limitations of proposed interventions [25,26]. Through fitness-for-work assessments, workplace adaptations, health surveillance activities, and collaboration with employers and treating specialists, they may therefore contribute to reducing disease-related disability and improving workforce participation among women affected by endometriosis. Moreover, as several studies report delays of many years between symptom onset and definitive diagnosis of endometriosis, OPs may often be requested to manage symptomatic workers without a formal diagnosis of the underlying condition [15,20,22,27,28]. In the absence of shared guidelines, knowledge gaps and inadequate understanding of endometriosis among OPs may lead to inappropriate requests for workplace modifications, unjustified restrictions, and inconsistent occupational recommendations, paradoxically worsening both occupational inclusion and health outcomes among affected workers.

The main objective of this study was therefore to characterize the understanding and management of endometriosis among Italian OPs by investigating their knowledge (awareness of the disease and relevant recommendations), attitudes (propensity toward specific management approaches), and practices (implementation of interventions directed at workers or workplaces), according to the Knowledge, Attitudes and Practices (KAP) framework. Knowledge, Attitudes and Practices (KAP) studies have been extensively employed to investigate how healthcare professionals approach emerging or insufficiently addressed health conditions [24,29,30,31,32,33,34,35]. By exploring knowledge gaps, risk perception, professional attitudes, and real-world practices, KAP studies may identify educational needs and inform future interventions. Ultimately, our findings may support the development of targeted educational and professional training initiatives for OPs, potentially improving occupational management and workplace inclusion of women affected by endometriosis.

## 2. Materials and Methods

### 2.1. Study Design

We designed the present study as a cross-sectional survey (see the Strengthening the reporting of observational studies in epidemiology, STROBE, checklist as Supplementary File S1) [36]. To reduce turnaround time, a web-based questionnaire was specifically developed (Google Forms; Google LLC.; Menlo Park, CA, USA) and shared between 1 April 2026 and 18 April 2026 through a closed discussion group whose application was officially limited to OP [29]. To maximize participation, a single reminder was distributed 7 days after the initial invitation (i.e., 8 April 2026). The authorization for sharing the study invitation and the appropriate link to the questionnaire was preliminarily requested from and granted by the principal investigator (MR). At the time of the survey, the group had 2213 unique members, but no information could be obtained regarding the number of members who were active participants.

The first page of the module included the full informed consent (Supplementary File S2), and participants were asked to provide consent for study participation through a mandatory dichotomous question (Yes/No). All participants agreeing with the informed consent were then requested whether they were or not a medical professional (Yes vs. No). In both cases, a negative answer led to the end of the questionnaire, with no further options for reviewing the remaining items, while a positive answer led to the full questionnaire.

### 2.2. Sample Size Calculation

As no preventive estimates on the previous professional experience of Italian medical workforce with endometriosis were available at the time of the study, we assumed as a reference the study from Roullier et al. [37] on French General Practitioners, which suggested that approximately 25% of physicians had adequate knowledge of endometriosis. Sample size estimation was performed manually using Cochran’s formula for a single population proportion. Assuming an expected proportion of 25%, a two-sided confidence level of 95% (α = 0.05; Z = 1.96), and an absolute precision (margin of error) of 5%, the minimum required sample size was calculated as follows:

N = 1.96^2^ × 0.25 × (1 − 0.25)/0.05^2^ = 3.8416 × 0.25 × 0.75/0.0025 = 288 (1)

**Figure A1:**
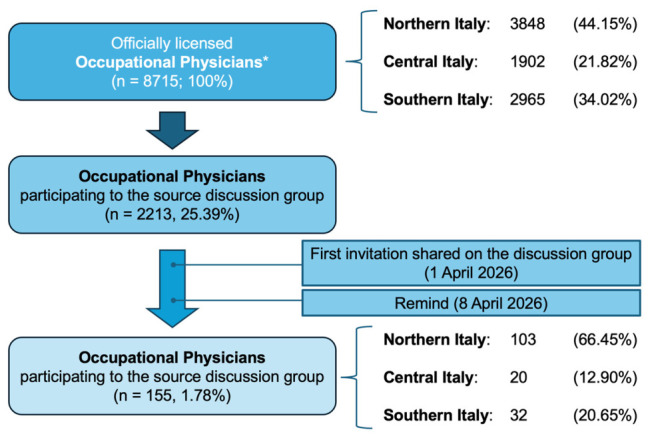
Flowchart of participating individuals. Starting from a total population of 8715 (i.e., the total number of officially licensed Italian occupational physicians), 2213 of them participating to the original discussion group, a total of 155 professionals were ultimately included in the final sample (i.e., 1.78% of total officially licensed occupational physicians and 7.00% of professionals participating to the original discussion group). Note: * = the total number of officially licensed Occupational Physicians can be retrieved from https://www.salute.gov.it/MediciCompetentiPortaleWeb/ricercaMedici.jsp; the repository is regularly updated; accessed on: 20 June 2026).

No finite population correction was applied, as the estimated sample size represented less than 5% of the target population of Italian OPs (i.e., 8715, Appendix A Figure A1), a threshold below which such correction is generally considered unnecessary.

### 2.3. Questionnaire

The questionnaire was designed as an anonymous one. Therefore, neither personal data (e.g., name, IP address, email address) nor any personal information not strictly related to the survey, and particularly clinical ones, were either requested, saved, or tracked. Even when dealing with demographic data, such information was requested in generic terms. No monetary compensation was offered to the participants, but they were guaranteed that at the end of the questionnaire a full explanation of all items would be provided, representing an educational opportunity on endometriosis.

As no specific questionnaire for endometriosis in occupational settings has been previously validated, the present instrument was specifically designed for this study following the blueprint of previous questionnaires employed in KAP studies on medical professionals [24,29,31,38,39]. The test–retest reliability of the questionnaire was preliminarily assessed through a survey on 15 healthcare workers (HCWs). Testers were asked to complete the questionnaire at two different points in time (T1 = 1 July 2025; T2 = 15 July 2025), and the paired comparison of all items was performed by calculation of Spearman’s rank test correlation coefficient (rho). Items having a rho > 0.800 were considered sufficiently consistent and were included in the final questionnaire. Beta testing questionnaires were not included in this study.

The final questionnaire included the following sections:

1. Main demographic data: age, gender, seniority, kind of medical background; the Italian region where the professional mainly worked and lived. Moreover, participants were inquired about their status as OP, i.e., if they were free practitioners or worked for a medical provider; whether they provided occupational health services to healthcare providers or not; whether they were authorized to practice as OPs through a full residency in Occupational Medicine, or by means of postgraduate training in Public Health or Legal Medicine, or through other qualifications recognized under Italian occupational health legislation [40,41]; if they had or not any background in psychiatry, internal medicine, and/or gynecology.
2. Knowledge Test. According to the medical applications of Health Belief Model (HBM) [24,42,43,44,45], knowledge status of a certain professional about a specific topic is key determinant of attitudes and behaviors. Therefore, a better knowledge status of endometriosis should be associated with more appropriate management of endometriosis cases. The knowledge section included 12 items, comprising 9 dichotomous questions and 3 multiple-choice questions (e.g., “Endometriosis is frequently associated with infertility”; TRUE) and 3 polytomous ones (e.g., “Endometriosis is usually diagnosed …” (a) between 12 and 14 years of age; (b) between 15 and 24 years of age; (c) between 25 and 34 years of age; (d) between 35 and 45 years of age; correct answer = c). The items were developed through an extensive review of the available literature on endometriosis. In order to ascertain whether the items of the knowledge were able to properly discriminate between participants with “strong” and “weak” understanding of endometriosis, the approach suggested by Möltner and Jünger was applied [46,47]. Briefly, correlation of each item of the knowledge test with the sum of all corrected answers was assessed through Spearman’s rank test; all questions with a rho ≥ 0.4 were included in a summary score (simplified Knowledge Score; GKS), which was calculated by assigning one point for each correct answer, while incorrect, missing, or “don’t know” responses received zero points. Moreover, consistently with the questionnaire from Roullier et al. [37], a series of conditions associated with endometriosis were presented, and participants were asked to rate their perceived occurrence among individuals affected by this condition (i.e., “Intense dysmenorrhea”, “Severe dyspareunia”, “Catamenial painful defecation”, “Chronic pelvic pain”, “Acute pelvic pain”, “Catamenial digestive disorders”, “Catamenial urinary function disorders”, “Menstrual cycle disorders”, “Fertility disorders”). Each item was rated in a 4-point Likert scale ranging from never, sometimes, often, to always.
3. Risk perception. According to the original report from Yates [48,49], perceived risk can be defined by the perceived probability of a certain event (F) and the expected consequences of that event (C). Participants were therefore requested to rate the perceived severity (C^END^) and the perceived frequency (F^END^) of endometriosis in Italian population by means of a fully labeled 5-point Likert scale (range: from “not significant”, score 1; to “very significant”, score 5). A cumulative Risk Perception Score (RPS) was therefore calculated as follows:

C^END^ × F^END^ = RPS (2)

4. Respondents were then asked to rate how difficult they perceived the management of endometriosis in Italian occupational settings compared to other chronic conditions, including: diabetes, allergic asthma, chronic dorsal pain, upper limb disease, heart disease, fibromyalgia, depression, epilepsy and migraine. All of the aforementioned statements were rated 1 (not difficult) to 10 (very difficult).
5. Attitudes. For the purposes of the present study, the attitude was defined as the tendency to evaluate a particular issue with some degree of favor or disfavor [48,49], and therefore reporting a certain attitude involved the expression of an evaluative judgment about a certain item. Respondents were therefore requested to rate through a full Likert scale 1 (very simple) to 5 (very complex) how they perceived the management of endometriosis due to a series of conditions (i.e., “Ergonomics of the workstation”, “Working hours”, “Workload”, “Work environment”, “Psychosocial factors”, “Interface between family and workplace”.
6. Practices. Participants were requested to report whether they had previously issued a restricted fitness-for-work appraisal (yes vs. no), then focusing on which interventions were actually implemented, if any: avoid night shift, and/or shift work, and/or exposure to extreme temperatures, and/or exposure to chemical agents, and/or front office duties, and/or implementation of increased number of work breaks.

### 2.4. Ethical Considerations

The informed consent guaranteed participants anonymity and confidentiality. Participation was voluntary, and the questionnaire was completed only by subjects who provided informed consent. As individual participants could not be identified from the collected data or the presented results, the study posed no foreseeable risk of harm or stigmatization to participants. Given its anonymous and observational design, the absence of patient-related clinical data, and the fact that it did not constitute a clinical trial, preliminary evaluation by an Ethics Committee was not required under Italian law (Gazzetta Ufficiale No. 76, dated 31 March 2008) [29].

### 2.5. Data Analysis

As a preliminary step, retrieved questionnaires were assessed for their completeness. Missing data were not expected, as all questionnaire items were mandatory within the Google Forms platform. Anyway, no imputation procedure was applied, and missing responses were handled through complete-case analysis. All continuous variables were reported as means with corresponding 95% confidence intervals (95% CIs), while categorical ones were reported as percent values. As a preliminary step, GKS, simplified GKS and RPS were normalized as percent values, and subsequently dichotomized according to the median value as high (>median) vs. low estimates (≤median). For analytical purposes, Likert-scale responses were dichotomized into lower to neutral and higher agreement categories.

Distribution of continuous variables was tested through the D’Agostino and Pearson K2 test. Given the limited sensitivity of normality tests to detect departures from normality, normality distribution was rejected for all *p*-values < 0.10, and variables were therefore compared through Mann–Whitney or Kruskal–Wallis tests for multiple independent samples, while their correlation was assessed through calculation of the Spearman’s rank correlation coefficient. On the other hand, a *p*-value ≥ 0.10 identified a normal distribution, and the variable were compared through the Student’s *t*-test for unpaired data or ANOVA, where appropriate, and their association was assessed by means of Pearson’s correlation test.

In univariate analysis, all variables were reported by the outcome variable of having or not previously issued any restricted fitness-to-work appraisal due to endometriosis, and the distribution was analyzed through chi-squared test with continuity correction. Internal consistency of the knowledge sections and its reliability were measured through calculation of the Cronbach’s alpha. Cronbach’s alpha (also known as rho-equivalent reliability) is a measure of how closely related a set of items are as a group. Although no universally accepted cut-off values do exist, a score ≥ 0.7 is considered for the acceptable reliability of the questionnaire.

A multivariable analysis was then performed by means of binary logistic regression, with calculation of adjusted odds ratio (aOR) and their respective 95% CI. Similarly to univariate analysis, the outcome variable was represented by having or not previously issued any restricted fitness-to-work appraisal due to endometriosis. Explanatory variables were all categorical variables on demographics and individual features of participants that at univariate analysis were significantly associated (i.e., *p* < 0.05) with outcome variables. Additionally, an exploratory hierarchical logistic regression analysis was performed according to the conceptual KAP framework. Variables were sequentially entered into four predefined blocks: demographic characteristics (Model I), knowledge-related variables (Model II), attitudes (Model III), and professional characteristics (Model IV), in order to explore the incremental contribution of each conceptual domain to the final model. To assess the robustness of the findings, two additional sensitivity analyses were performed. First, a conventional maximum-likelihood logistic regression model was fitted including all candidate demographic, professional, knowledge-related, and educational variables, irrespective of their univariate statistical significance. Second, the same fully adjusted model was re-estimated using Firth’s penalized logistic regression to reduce potential small-sample and sparse-data bias. Firth’s penalized logistic regression was adopted because it reduces small-sample bias and provides finite parameter estimates in the presence of complete or quasi-complete separation.

Statistical analyses were performed by means of IBM SPSS Statistics 31.0 for Macintosh (IBM Corp., Armonk, NY, USA), GraphPad Prism version 11.0.0 for Macintosh, (GraphPad Software, Boston, MA, USA), R (version 4.6.0) [19] and Rstudio (version 2026.04.0 Build 256; Rstudio, PBC; Boston, MA, USA) software by means of the packages epiR (version 2.0.62), fmsb (version 0.7.5), ggpubr (version 1.0.0), logistf (version 1.26.1), broom (version 1.0.13), stats (version 4.6.0).

## 3. Results

### 3.1. Characteristics of the Study Population

As shown in Table 1, 155 OPs eventually participated to the survey, corresponding to 1.78% of all licensed Italian OPs: as the parent group included a total of 2213 members, a 7.00% response rate could be calculated (Appendix A Figure A1). Because the survey was administered through a web-based platform requiring completion of all mandatory items before submission, no partially completed questionnaires were available for analysis. Owing to the anonymous design of the survey, it was not possible to determine the number of individuals who initiated the questionnaire but did not complete the submission process.

The mean age of respondents was 43.75 years (95% CI 42.27 to 45.24), with a mean seniority of 17.05 years (95% CI 15.45 to 18.64). Male participants accounted for 58.71% of the sample. Most respondents were from Northern Italy (66.45%), followed by Southern (20.65%) and Central Italy (12.90%), suggesting a substantial oversampling of professionals from northern regions compared to officially available data (i.e., 3848, 1902, and 2965 for Northern, Central, and Southern Italy, respectively; chi squared test 30.66, df = 2; *p* < 0.001).

Most participants worked as OPs after having completed a full residency in Occupational Medicine (87.74%), whereas smaller proportions reported training in Hygiene and Public Health (11.61%) or Legal Medicine (0.65%). Overall, 67.10% worked as OPs for healthcare providers, and 74.84% reportedly were free practitioners. Most respondents considered themselves adequately informed about endometriosis (78.06%). However, when focusing on the main source of medical information on endometriosis, most of participants reported that medical school still represented their main source of information (67.74%), followed by internet-based resources (31.61%), gynecologist colleagues (30.32%), and CME activities (18.71%). Notably, 10.97% of respondents reported that guidelines specifically addressing the occupational management of endometriosis do currently exist. As such guidelines currently do not exist, this finding may be interpreted as a proxy for a certain familiarity of participants with endometriosis. Not coincidentally, any previous professional experience with women affected by endometriosis was reported by 81.29% of participants. The majority of them did previously formulate conditioned fitness-to-work appraisals that included restriction to specific risk factors (65.81%), and more precisely: increasing the number of breaks during the shift (50.32%), followed by removal of night shifts (30.32%), and more in general of the shift work (23.23%). Participating occupational physicians also reported to recommend the avoidance of exposures to front office duties (11.61%), and extreme temperatures (9.68%), while only 1.94% recommended the avoidance of exposure to chemical agents.

### 3.2. Knowledge of Endometriosis

Full details of knowledge tests are reported in Table 2. Briefly, the internal consistency was estimated to Cronbach’s alpha = 0.900: this finding supports the internal coherence of the knowledge construct and suggests that the included items adequately captured a common underlying domain.

Despite the high proportion of participants self-reporting adequate knowledge of endometriosis (78.06%), substantial deficiencies emerged in epidemiological and diagnostic domains, suggesting a discrepancy between perceived and objective knowledge. Moreover the proportion of correct answers varied substantially across individual items. Nearly all participants correctly recognized endometriosis as the presence of endometrium-like tissue outside the uterus (100.0%), and most were aware of its association with infertility (83.2%), psychiatric comorbidities such as depression (91.0%), and the aims of treatment (81.9%).

Conversely, knowledge gaps emerged regarding epidemiology and diagnosis. Only 26.45% correctly identified the prevalence of endometriosis among women of reproductive age, while 31.61% correctly recognized that transvaginal ultrasound is not the diagnostic gold standard. Similarly, only 34.84% acknowledged the strong familial predisposition of endometriosis.

**Figure A2:**
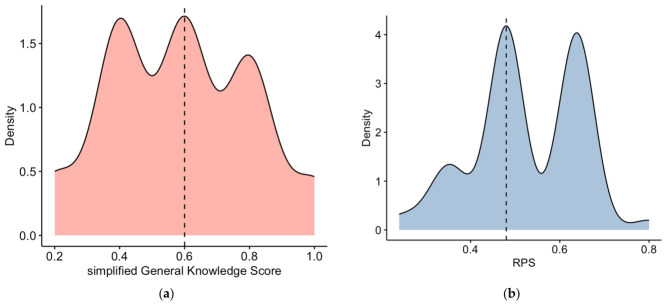
Distribution of: (**a**) simplified General Knowledge Score (mean: 58.71%; 95% confidence interval 55.25 to 62.16; K2 test = 8.968, *p* = 0.011); (**b**) Risk Perception Score (mean 52.52%; 50.64 to 54.40; K2 test = 3.736, *p* = 0.154). Dotted lines represent median values.

General Knowledge Score (GKS) was eventually calculated on the whole of 12 items, being estimated to 64.14% (95% 62.21 to 66.07). The cumulative score was significantly associated with the following items, which showed the strongest discriminatory capacity across respondents and were therefore included into the sGKS (sum score = 58.7%; 95% CI 55.3–62.2): considering early menarche as a risk factor for endometriosis (rho = 0.338, *p* < 0.001), acknowledging endometriosis as associated with infertility issues (rho = 0.312, *p* < 0.001), the role of the abnormalities of pelvic musculature (rho = 0.172, *p* = 0.033), and the diagnostic issues related to transvaginal ultrasound (rho = 0.166, *p* = 0.040) (Appendix A Figure A2a).

**Figure 1:**
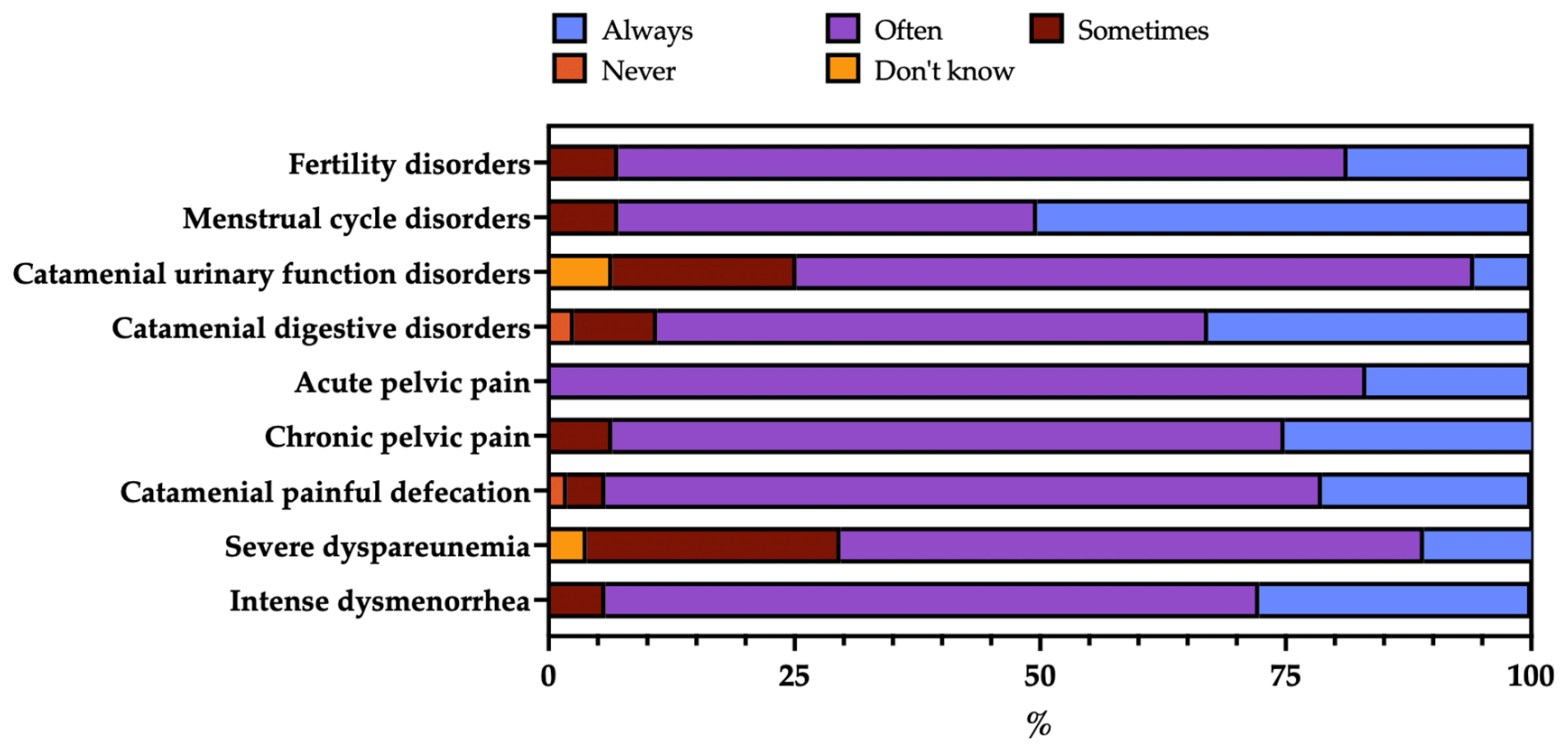
Perceived frequency of the main clinical and occupational issues experienced by workers affected by endometriosis, as reported by participating occupational physicians. Bars represent the percentage of respondents indicating each condition among the most commonly encountered problems during occupational health management.

Participants were then inquired about which clinical features they considered associated with endometriosis (Figure 1). The whole of respondents identified acute pelvic pain as either always or often reported by women with endometriosis (100%), followed by intense dysmenorrhea, and catamenial painful defecation (both conditions, 94.19%), chronic pelvic pain (93.55%), disorders of menstrual cycles and fertility disorders (both conditions, 92.90%), catamenial disorders affecting digestive (64.52%) and urinary function (74.83%), while severe dyspareunia was reported by 70.32% of participants.

### 3.3. Risk Perception

**Figure 2:**
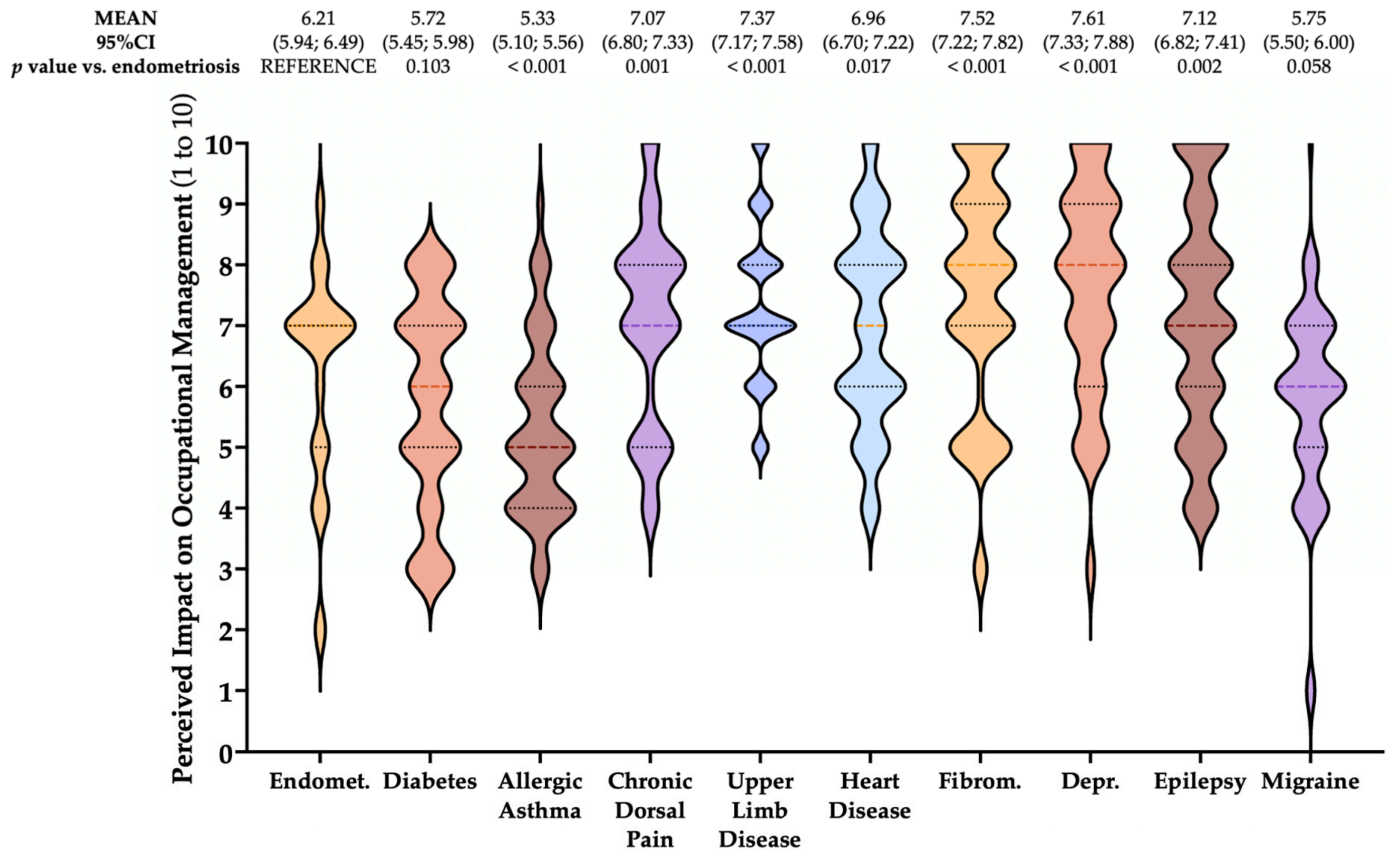
Violin plots showing the perceived difficulty in the managing of endometriosis in the workplaces as reported by participants through a synthetic score 1 (no concern) to 10 (very high concern), compared to other conditions (diabetes, allergic asthma, chronic dorsal pain, upper limb disease, heart disease, fibromyalgia, depression, epilepsy, and migraine), according to participating occupational physicians. The width of each violin represents the distribution density of responses. Horizontal dashed lines indicate the median and interquartile range. Mean scores (95% confidence intervals) are reported above each violin; all comparisons were performed using the Kruskal–Wallis test.

Focusing on the factors participating in the definition of perceived risk of endometriosis, 70.97% of participants acknowledged this condition as severely impacting on the health of affected individuals, and 55.48% as relatively frequent, for an eventual RPS of 52.52% (95% CI 50.64 to 54.40) (Appendix A Figure A2b). As shown in Figure 2, participants generally perceived endometriosis as a clinically relevant condition, rated with a mean severity score of 6.21 (95% CI 5.94–6.49) (potential range, 0 to 10). Several chronic disorders were perceived as significantly more difficult to manage in daily practice than endometriosis, including chronic dorsal pain (7.07, 95% CI 6.80 to 7.33; *p* = 0.001), upper limb disorders (7.37, 95% CI 7.17 to 7.58; *p* < 0.001), ischemic heart disease (6.96, 95% CI 6.70 to 7.22; *p* < 0.001), fibromyalgia (7.52, 95% CI 7.22 to 7.82; *p* < 0.001), depression (7.61, 95% CI 7.33 to 7.88; *p* < 0.001), and epilepsy (7.12, 95% CI 6.82 to 7.41; *p* = 0.002). Conversely, diabetes and migraine were considered as similar to endometriosis in terms of perceived severity (5.72, 95% CI 5.45 to 5.98; *p* = 0.103, and 5.75, 95% CI 5.50 to 6.00; *p* = 0.058).

### 3.4. Perceived Barriers to Workplace Management

**Figure 3:**
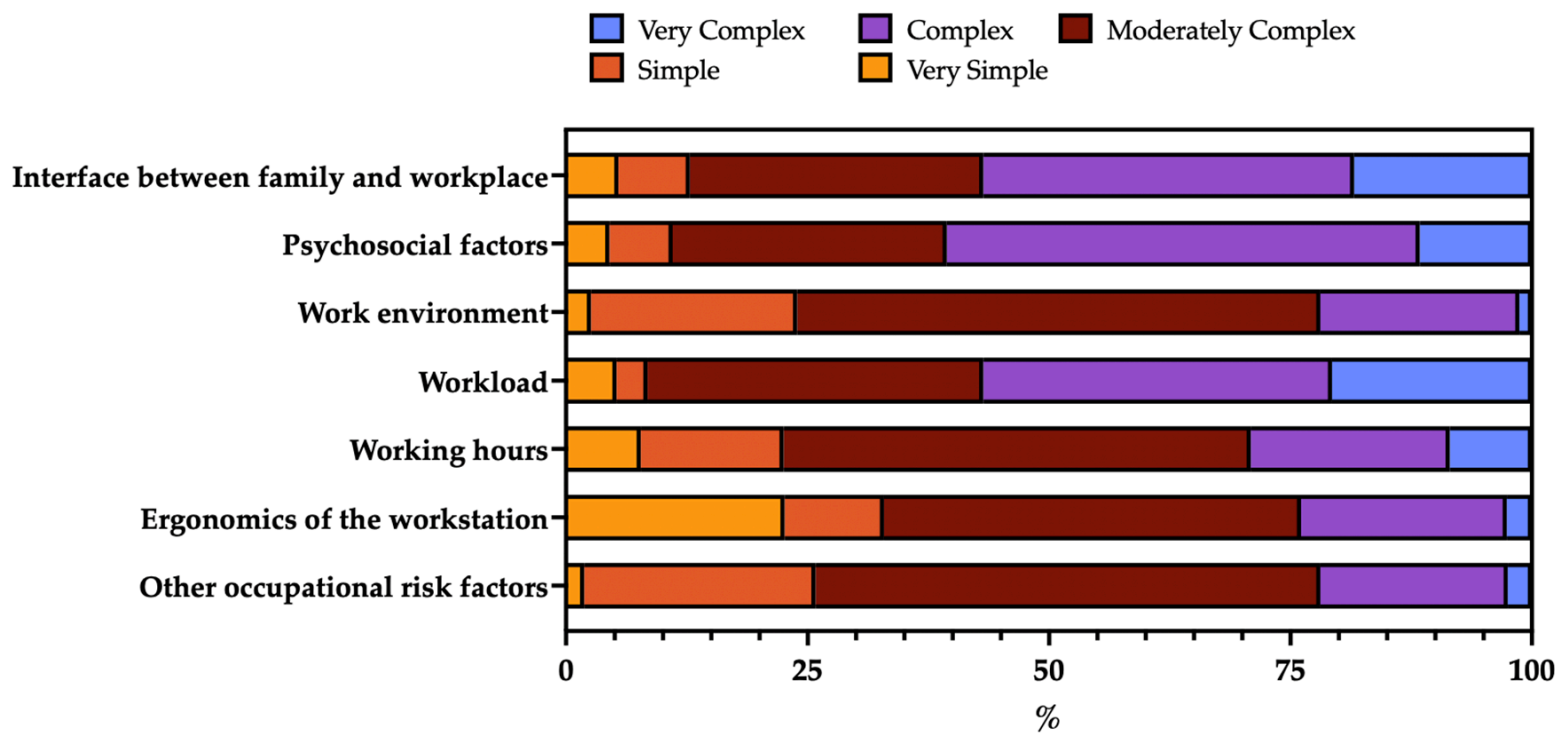
Perceived barriers to the occupational management of workers affected by endometriosis, according to participating occupational physicians. Stacked bars represent the proportion of respondents rating each occupational domain (family–work interface, psychosocial factors, work environment, workload, working hours, workstation ergonomics, and other occupational risk factors) as very simple, simple, moderately complex, complex, or very complex to manage.

Barriers to workplace management of patients affected by endometriosis are reported in Figure 3. Briefly, managing of psychosocial factors (i.e., work-related stress resulting from poor work design, organization and management) was considered the most significant barrier, being acknowledged as either complex or very complex by 60.65% of participants, followed by workload and interface between family and workplace (56.77%), managing of working hours (29.03%), ergonomics of the workstation (23.87%), and more generally by characteristics of the work environment (21.94%).

### 3.5. Factors Associated with Conditional Fitness-to-Work

In univariate analyses (Table 3), physicians issuing conditioned fitness-for-work judgments were more frequently male (74.51% vs. 28.30%; *p* < 0.001), older (44.88, 95% CI 42.97 to 46.80 vs. 41.58, 95% CI 39.33 to 43.84 years, *p* = 0.036), and had longer professional seniority (18.37, 95% CI 16.35 to 20.39 vs. 14.49, 95% CI 11.99 to 16.99 years, *p* = 0.022) than other professionals. Interestingly, the proportion of OPs from central/southern Italy was significantly higher among participants having issued any conditioned fitness-for-work than among other professionals (40.20% vs. 20.75%, *p* = 0.024). Regarding their professional background, the proportion of participants with a full residency in Occupational Medicine (87.25% vs. 88.68%) and working as free practitioners (76.47% vs. 71.70%) was similar among OPs having issues any occupational restriction due to the diagnosis of endometriosis (*p* = 1.000 and *p* = 0.650. respectively); no significant differences were found between the two groups in the proportion of participants reporting previous training in gynecology (13.73% vs. 9.43%), psychiatry (6.86% vs. 11.32%), or internal medicine (66.67% vs. 64.15%). On the contrary, the proportion of participants reporting that they provided occupational health services to a healthcare provider was more than twice as high among those who had previously issued a conditioned fitness-for-work appraisal than among those who had not (83.33% vs. 35.85%, *p* < 0.001). While no differences were identified regarding self-reported knowledge of endometriosis (78.43% vs. 77.36%, *p* = 1.000), OPs reporting any conditioned fitness-to-work appraisal due to endometriosis more frequently scored a sGKS above the median (41.2% vs. 15.1%; *p* = 0.002) than remaining participants.

Regarding the proportion of participants scoring a RPS higher than median value, it was highest among professionals having issued any restricted fitness-to-work appraisal of women affected by endometriosis (47.06% vs. 33.96% among other participants), but the difference was not significant (*p* = 0.164).

Focusing on the source of information, having benefited of CME interventions on endometriosis, having acquired information on this condition from colleagues, and having benefited of internet-based resources were more frequently reported by participants having previous issued work restrictions than by other participants (27.45% vs. 1.89%, *p* < 0.001; 40.20% vs. 11.32%, *p* < 0.001; and 38.24% vs. 18.87%, *p* = 0.023, respectively). On the contrary, reliance on medical school training was less frequently reported among participants having issued restricted fitness-to-work due to the endometriosis (53.92% vs. 94.34%, *p* < 0.001).

### 3.6. Attitudes and Perceptions Towards Endometriosis and Its Management

**Figure A4:**
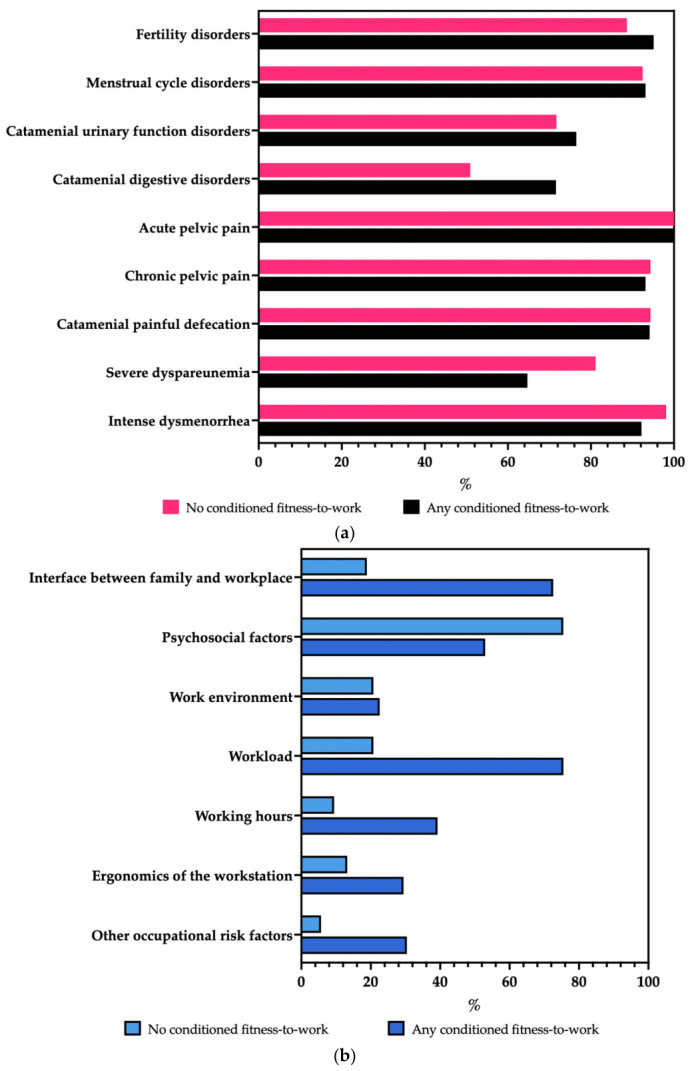
Distribution of symptoms associated with endometriosis (**a**) and main barriers for the management of endometriosis in the workplaces (**b**) as reported by 155 occupational physicians participating to the present survey.

Perceived difficulty in the managing of endometriosis in the workplaces was similarly rated by participants having (6.33, 95% CI 5.98 to 6.68) and having not issued any conditioned fitness-to-work appraisal (5.98, 95% CI 5.52 to 6.44; U = 3106.00; *p* = 0.105) (Table 4). Interestingly enough, when focusing on the clinical features that participants considered as associated with endometriosis, similar proportions were reported in both groups of respondents, with the notable exception catamenial digestive disorders, more frequently associated with having issued any fitness-to-work restriction (71.57% vs. 50.94%, *p* = 0.018), and severe dyspareunia, that was more frequently considered by respondents who had not reported any restriction (64.71% vs. 81.13%, *p* = 0.053) (Appendix A Figure A4).

Main perceived barriers to workplace management of endometriosis were also assessed, and having issued any conditioned fitness-to-work appraisal was significantly associated with most of reported factors, including the ergonomics of the workstation (29.41% vs. 13.21%, *p* = 0.041), working hours (39.22% vs. 9.43%, *p* < 0.001), workload (75.49% vs. 20.75%, *p* < 0.001), and the interface between family and workplace (72.55% vs. 18.87%, *p* < 0.001). Conversely, participants having issued any restricted fitness to work less frequently considered complex and/or very complex the management of psychosocial factors (52.95% vs. 75.47%, *p* = 0.011) (Appendix A Figure A4).

Distribution of perceptions regarding clinical features and occupational issues of endometriosis were also assessed by taking into account having or not continuous medical education (CME) activities as the main source of information on endometriosis, having or not a full residency in Occupational Medicine, working or not as occupational physician (OP)s for any healthcare provider. Results are reported in Appendix A Table A1 in full details. In brief, participants who had attended CME activities on endometriosis were significantly more likely to identify working hours as a barrier to the occupational management of workers affected by endometriosis (48.28% vs. 24.60%, *p* = 0.021). Conversely, they were significantly less likely to recognize psychosocial risk factors as a relevant barrier (34.48% vs. 66.67%, *p* = 0.003). No other differences were identified.

### 3.7. Multivariable Analysis

In the end, multivariable analysis included as the outcome variable having issued any restricted fitness-to-work appraisal of women affected by endometriosis, while the explanatory variables were implemented: being of male gender, age, seniority, being from Northern Italy, reporting a sGKS above median value, having as a source of information medical school, continuous medical education, colleagues, internet, and working as occupational physician in healthcare settings.

**Figure 4:**
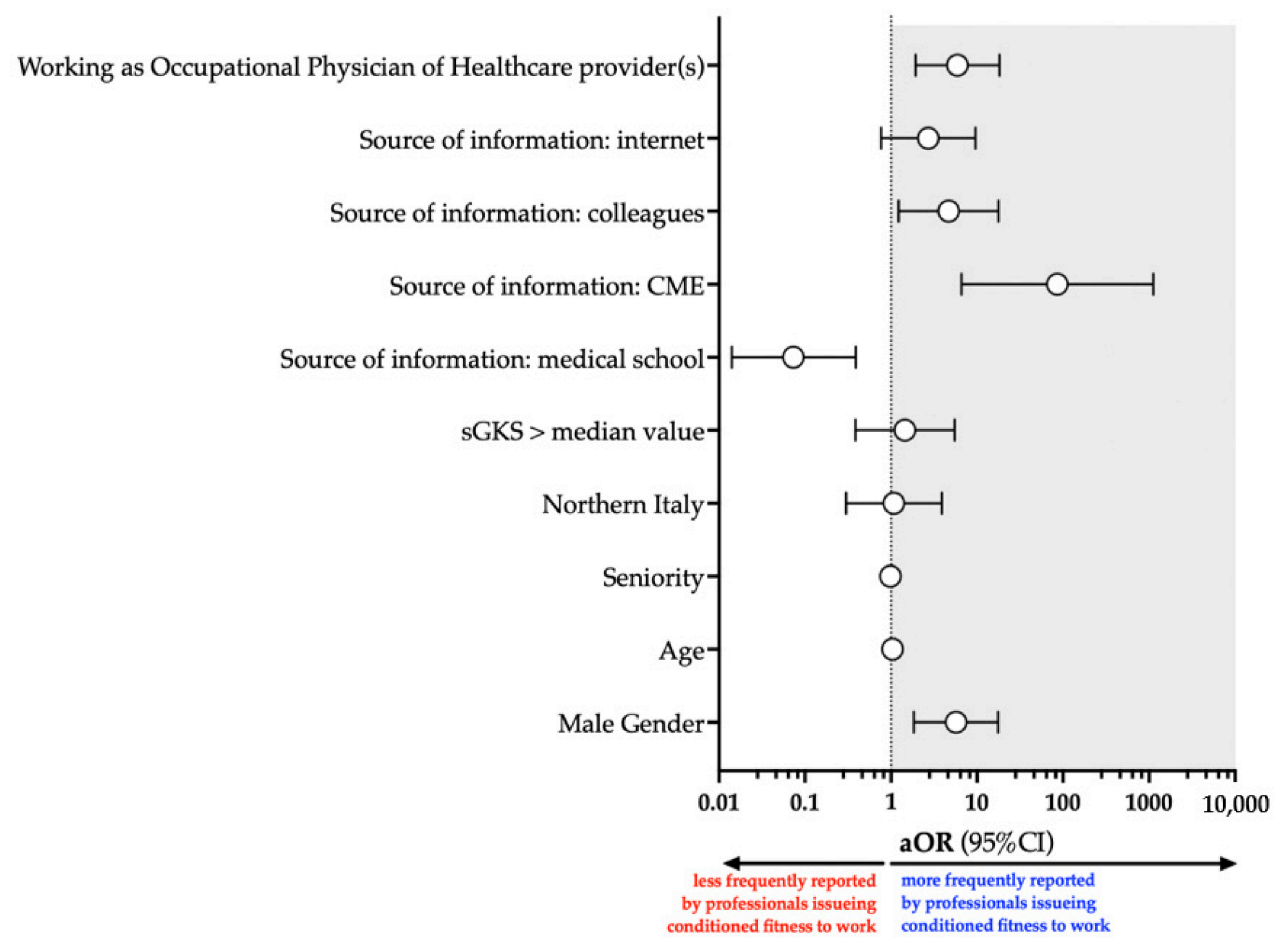
Results of multivariable analysis on the outcome variable of having issued conditioned fitness to work (aOR = adjusted odds ratio, 95% CI; CME = continuous medical education; sGKS = simplified General Knowledge Score).

As reported in Figure 4 (see also Appendix A Table A2), male gender (aOR 5.685, 95% CI 1.839 to 17.579), having as a source of information on endometriosis continuous medical education activities (aOR 85.720, 95% CI 6.595, 1114.208), colleagues (aOR 4.669, 95% CI 1.226 to 17.774), and working as OPs for any healthcare provider (aOR 5.909, 95% CI 1.923 to 18.162) were positively associated with having issued any previous restricted to work appraisal due to endometriosis.

On the contrary, having medical school as the sole source of information on endometriosis was negatively associated with the outcome variable (aOR 0.073, 95% CI 0.014 to 0.388).

An exploratory hierarchical logistic regression was performed according to the conceptual KAP framework by progressively introducing demographic characteristics (Model I), knowledge-related variables (Model II), attitudes (Model III), and professional characteristics (Model IV) (See Table 5). Overall, the sequential inclusion of additional variable blocks did not materially modify the associations observed in the baseline model. Male gender remained consistently associated with a higher likelihood of having previously issued a conditioned fitness-for-work appraisal across all models (OR ranging from 5.584 to 7.172). Conversely, neither the simplified sGKS (final model: OR 0.590, 95% CI 0.135–2.588) nor RPS (OR 0.876, 95% CI 0.212–3.624) independently predicted the outcome. In the final model, working as an OP for healthcare providers remained strongly associated with the previous issuance of conditioned fitness-for-work appraisals (OR 9.546, 95% CI 2.292–39.761), whereas relying on undergraduate medical education as the main source of information was inversely associated with this outcome (OR 0.048, 95% CI 0.007–0.322). Overall, the hierarchical analysis confirmed the robustness of the primary multivariable findings.

### 3.8. Sensitivity Analisys

**Figure 5:**
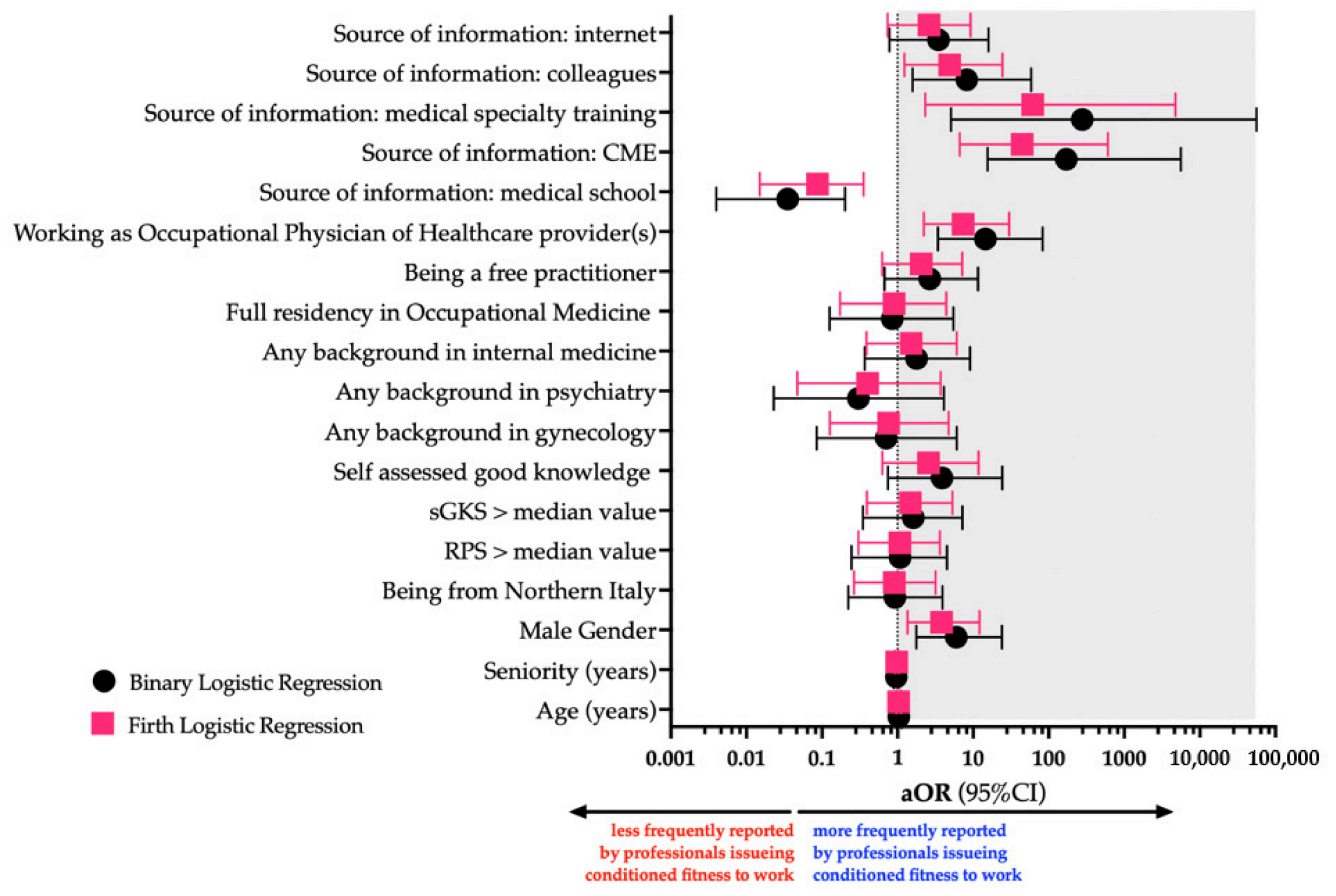
Results of sensitivity analysis. To assess the robustness of the findings, a conventional maximum-likelihood logistic regression model was fitted including all candidate demographic, professional, knowledge-related, and educational variables, irrespective of their univariate statistical significance. The same fully adjusted model was re-estimated using Firth’s penalized logistic regression to reduce potential small-sample and sparse-data bias (aOR = adjusted odds ratio, 95% CI; CME = continuous medical education; sGKS = simplified General Knowledge Score; RPS = risk perception score).

Two sensitivity analyses were subsequently performed. In the fully adjusted conventional logistic regression model, the direction of the main associations was broadly consistent with that observed in the primary analysis, although several estimates were characterized by wide confidence intervals. Re-estimation of the same model using Firth’s penalized logistic regression attenuated some of the largest effect estimates and improved their stability, while leaving the overall direction of the associations substantially unchanged (Figure 5, Appendix A Table A2).

## 4. Discussion

### 4.1. Summary of Main Results

Through the present cross-sectional study, we provide some insights into how Italian OPs perceive and manage endometriosis in workplace settings. Overall, a total of 155 professionals participated into the survey, that is nearly half of the targeted sample size (n = 288, 53.81%), with a relatively low response rate (7.00%). Moreover, the eventual sample over-represented professionals from Northern regions (66.45% vs. 44.15% from official estimates), urging for the quite cautious appraisal of main results.

Participants appeared to report a relatively high degree of familiarity with endometriosis, as 78.06% of respondents considered themselves adequately informed about the disease, and 81.29% of them did previously manage any patient affected by endometriosis. Still, endometriosis was not perceived as relatively relevant occupational health issue: not only the mean RPS was estimated to 52.52% (95% CI 50.64 to 54.40), but also the perceived difficulty in its managing across the workplaces was rated as lower than that for other chronic conditions such as depression, fibromyalgia, ischemic heart disease, etc.

Objective knowledge appeared affected by substantial knowledge gaps, as only 26.45% correctly estimated disease prevalence, 34.84% correctly identified its familial aggregation, and 31.61% recognized laparoscopy with histological confirmation as the diagnostic gold standard. On the other hand, most participants were properly aware that official Italian guidelines for occupational management of endometriosis do not exist. At the time of the survey, official recommendations were exclusively focused on clinical diagnosis and treatment, with limited value for OPs and their daily practice [49]. Therefore, as this false statement was only reported by 11% of participants, we can indirectly argue that the vast majority of respondents were aware of this important gap in the current evidence base.

In our analyses, we included having previously issued any restricted fitness-for-work appraisal due to endometriosis as the outcome variable. Within the Italian legal framework [50], a prescription is a legally binding measure issued by the competent OPs that specifies the conditions, limitations, or restrictions under which a worker may safely perform a given job [40,51]. Its purpose is to protect the worker’s health while allowing continued employment whenever possible. In the context of endometriosis, the need to issue a prescription may be interpreted as reflecting the inability to achieve adequate protection through structural or organizational workplace modifications alone [51]. In other words, it may represent a measure of last resort, adopted when the work environment has not been sufficiently adapted to accommodate the needs of affected workers. For this reason, it should not be interpreted as either an inherently positive or an inherently negative practice. Rather, it may be viewed as a proxy for the inability of the occupational system to adequately adapt to the needs of an individual worker affected by endometriosis, the preferable option whenever feasible, as more likely to ensure an inclusive, effective and long-lasting management of workers’ health and safety [50]. Eventually, having issued any previous fitness-to-work restriction was associated with previous participation in CME activities, having acquired information on endometriosis from colleagues rather than other educational options, and working for any healthcare provider, and such results were otherwise confirmed by two distinctive models of sensitivity analyses.

### 4.2. Generalizability of Main Data and Implication for Daily Practice

Endometriosis is a common condition among women of reproductive age [1,4,5], with documented impact on personal [1,2,5,6,7,8] and social life of affected individuals [9,10], with a considerable impact on the quality of life [3,9,11,12]. Although reproductive age largely overlaps with working age, the engagement of OPs in the management of affected individuals appears both necessary and unevenly documented in the international scientific literature [16,21,22,23]. Respondents in fact demonstrated a moderate level of knowledge regarding the condition and a similarly improper awareness of its clinical relevance, with substantial gaps emerging on epidemiological aspects, diagnostic pathways, and familiarity with occupational management guidelines. More precisely, one of the most relevant findings was the discrepancy between self-perceived knowledge and objective knowledge indicators. Even though most participants considered themselves adequately informed about endometriosis, performance on several knowledge items revealed important limitations.

While, at our knowledge no similar studies have been performed among OPs, present results can be considered as consistent with previous surveys conducted among other categories of healthcare professionals [37,52,53], suggesting that endometriosis remains insufficiently covered during undergraduate and postgraduate medical training, urging for a global call to action [54]. For example, Roullier et al. [37] following the publication of updated French guidelines on endometriosis (2017), performed a KAP study on 102 General Practitioners (GPs) from the Picardie region of France, documenting a substantial lack of self-assessed understanding of this condition. Not only less than 20% of participants were aware of existing national guidelines, but self-assessed understanding of endometriosis for daily routine clinical practice was considered as sufficient by no more than 25% of enrolled GPs. Moreover, most participants exhibited significant knowledge gaps on signs of symptoms and their diagnostic value. In this regard, it should be borne in mind that endometriosis is often diagnosed several years after symptom onset, with definitive diagnosis frequently occurring during the third or fourth decade of life. Resulting diagnostic delay may place OPs in a strategic position for the eventual recognition of affected workers, eventually improving their quality of life [15,20,22,27,28]. These results were quite consistent with a similarly designed study on 185 GPs from New Zealand, as only 52% of them considered themselves to know enough about endometriosis for daily practice, and the eventual awareness of this condition was eventually rated as quite unsatisfying [53,54]. Eventually, a KAP study from Saudi Arabia on 215 nurses from Al-Jouf region documented a large proportion of low knowledge, with only 6% of participants achieving high scores, particularly among professionals with high educational attainment, greater seniority, and direct exposure to patient care [52].

Still, it is important to acknowledge that the above-mentioned studies are only partially comparable with a survey conducted among OPs, for several reasons. First of all, the competence domains of OPs are usually quite distinctive from those of GPs [41]. In countries where this professional figure is fully integrated into the occupational health regulatory framework [25,26], occupational physicians are inherently required to address a specific issue, namely the maintenance of workers’ health within the context of continued employment and job retention [55,56,57]. Conversely, studies conducted among GPs, and to some extent the survey by Elsharkawy et al. [52] involving nursing personnel, primarily focused on diagnostic and therapeutic aspects of endometriosis rather than its occupational implications. A second substantial difference with aforementioned studies is represented by the sheer number of OPs compared to other medical professionals, and particularly GPs. According to available figures, the total workforce of Italian GPs can be estimated to around 40,000 professionals [58,59], that is nearly five times the pool of officially licensed OPs (i.e., 8715 by 20 June 2026). In other words, albeit the recruited sample was relatively small, and certainly smaller than required by preventive sample size calculation, not only the number of respondents was similar to the studies from Roullier et al. [37] and from from New Zealand [53,54], but the proportion of participating professionals was indeed noticeable when taking into account the total number of licensed Italian OPs, encompassing therefore 1.78% of all Italian professionals, i.e., up to around 2% of the whole of working population. Moreover, when compared with available studies on KAP of GPs on endometriosis, our results provide data that can share a unique perspective on endometriosis. As a part of their professional duties, OPs are expected to balance disease-related limitations, workers’ well-being, and the feasibility of workplace accommodations aimed at preserving participation in the active workforce. In this regard, the lack of specific guidelines for occupational management of endometriosis is more than noteworthy. Most of international guidelines on endometriosis focus on aspects such as diagnosis and clinical management, of certain interest for GPs [60,61,62,63]. On the contrary, available documents only provide scarce recommendations on workplace adjustments or avoidance of occupational exposures that may aggravate symptom burden. In the absence of standardized approaches, management decisions are likely influenced by individual experience and professional judgment, eventually providing some explanations on reported prescription. While the increased number of work breaks (50.3%) may guarantee some sort of relief to patients affected by chronic pain and fatigue, the importance of avoiding night and shift work (30.3% and 23.2%, respectively), as well as the avoidance of the exposure to office duties (11.6%) and extreme temperatures (9.7%) may be of limited significance. Knowledge gaps were likely due to the lack of specific post-graduate formation on this specific topic, mostly associated with university courses during the medical school (67.7%), with a large share of respondents relying on personal interaction with colleagues of other specialties, and particularly gynecologists. The very low proportion of respondents claiming to have received a specific formation during the post-graduation formation may be of particular interest, as it stresses that participants were likely inclined to frame endometriosis management according to a model more akin to general practice than to the occupational health paradigm.

A significant message that can be shared from our data is that participants extensively acknowledged endometriosis as a potentially significant condition. Unfortunately, actual understanding of participants was affected by substantial knowledge gaps encompassing not only how detrimental endometriosis may be for the quality of life and for the quality of working life of female workers, but also how to properly menage endometriosis in daily practice. Not coincidentally, participating OPs considered its management on the workplaces less complicated than that of several other chronic disorders, including depression, fibromyalgia, epilepsy, and cardiovascular disease. Interestingly enough, no substantial differences were spotted between participants having a background including a full residency in Occupational Medicine and professionals with a post-degree formation in Hygiene and Public Health and/or Legal Medicine (see also Appendix A Table A1). In other words, as participants substantially shared the general appraisal of endometriosis as a significant medical condition, but also the whole of knowledge gaps, the latter should be acknowledged as resulting from general flaws of educative programs, that could be filled by a revision of core curricula of post-degree formation and by increasing the availability of specifically tailored CME activities.

The general underscoring of endometriosis, both in general, as stressed by the RPS estimate, and compared to other chronic conditions, could be somewhat surprising, given that, unlike endometriosis, many of the aforementioned conditions benefit from validated preventive interventions and consolidated occupational management strategies, which usually permit continued employment and the maintenance of safe working conditions [29,64,65,66]. In particular, the increasingly aging workforce did lead to several guidelines on the accommodations for cardiovascular conditions [67,68,69]. This may reflect the persistent under-recognition of the broader functional and psychosocial burden associated with endometriosis, despite growing evidence documenting its substantial impact on daily activities and work productivity. As recently stressed by Ellis et al. [54], significant gaps remain in the proper understanding of this condition, disproportionately low when compared to the pain of patients, discomfort due to the long delays in diagnosis, the ineffectiveness of common treatment options, and the substantial economic burden borne by individual patients. Although this issue is inherently difficult (if not impossible) to address through an evidence-based perspective, it cannot be excluded that underlying knowledge gaps lead competent OPs to improperly perceive workers affected by endometriosis as “complex” or intrinsically problematic individuals, with insufficient attention being paid to the impact of their underlying condition on work ability and occupational functioning [37,52,54].

Another potential explanation for the widespread tendency to underestimate the burden of endometriosis is hinted by the significant role acknowledged by participating OPs to psychosocial factors and particularly to the interaction between family and occupational responsibilities. Alongside workload, these factors were perceived as the most challenging aspects of workplace management for individuals affected by endometriosis. As stressed by European Agency for Safety and Health at Work (EU OSHA), psychosocial risks factors arise from poor work design, organization and management, as well as from poor social context of work [70]: resulting in negative psychological, physical and social outcomes, their interplay with an underlying condition such as endometriosis may be significant even in settings that could be considered at relatively low risk for the large majority of workers [6,11,14]. Even though managing work-related psychosocial risks is not just a moral obligation and a good investment for employers but also represents a legal imperative in several legal framework (e.g., Framework Directive 89/391/EEC for EU countries), stressing the pivotal role of OPs [71,72,73], both employers and OPs usually perceive these conditions as particularly complicated to be properly managed [71,73]. Within the specific context of Italian occupational medicine, it cannot be excluded that some OPs may improperly perceive the management of these issues as partially exceeding their professional remit [72,73], considering them to fall primarily within the legal and moral responsibilities of employers [72,74].

The interplay of knowledge gaps and the acknowledgement of psychosocial risk factors among the main determinants of barriers for the proper occupational management of endometriosis provides a likely explanation for the very high proportion of conditioned fitness to work statements reported by study participants. As previously stressed, the issuance of a conditioned fitness-for-work appraisal may represent an alternative to broader organizational or structural interventions [50]. This is of particular significance when dealing with an underlying condition (such as endometriosis) that, while compromising the overall quality of life of affected individuals, not consistently requests the implementation of specific occupational restrictions. From this perspective, the factors that were independently associated, either positively or negatively, with the issuance of conditioned fitness-for-work appraisals in the multivariable analysis may be interpreted in light of both the Italian occupational health framework and more general considerations. Undergraduate medical education on endometriosis, which is typically focused on diagnostic and therapeutic aspects, may not necessarily lead physicians to perceive this condition as a complex occupational issue requiring specific workplace management strategies. Conversely, previous attendance at dedicated educational initiatives on endometriosis, within a discipline where this topic remains comparatively underrecognized, may reflect a certain degree of self-selection, potentially resulting in an oversampling of professionals who are particularly sensitive to this issue and therefore more inclined to adopt precautionary approaches, including the issuance of conditioned fitness-for-work appraisals. Likewise, the independent association observed among OPs working in healthcare settings may plausibly be explained by the predominantly female composition of the healthcare workforce, increasing the likelihood of encountering workers affected by endometriosis, but also by the generally more developed occupational health policies and greater availability of workplace accommodations that characterize healthcare institutions, particularly within the Italian public sector.

### 4.3. Limits

The present study has several limitations. First, the cross-sectional design prevents causal inference [75,76]. Therefore, although we identified a series of conditions significantly associated with a certain outcome (in this case, having issued previous fitness-to-work restrictions), no causal relationship can be assumed, limiting our appraisal to the acknowledgement of a significant association with conditions such as being of male gender having participated to CME activities, relying on colleagues for being informed on endometriosis, employment as OPs of healthcare providers, and relying exclusively on undergraduate medical education.

Second, the present study shares the whole of implicit limits of similarly designed Internet-based surveys [31,77], and particularly the extensive “self-selection” of participants, leading to the oversampling of certain sub-groups. The extensive proportion of subjects familiar in sharing personal information through internet and social media as well as of individuals exhibiting a proactive attitude or greater knowledge about the assessed topic may lead to the overestimation of the actual understanding of the targeted population. Even though, the potential self-selection of the participants has been somewhat mitigated by targeting a very specific and therefore quite homogenous subgroup of medical professionals, i.e., OPs, generalization of main results should be only cautiously considered. On the other hand, having our study relied on a closed discussion group, whose participation was strictly limited to licensed OPs, we can rule out that some of the respondents did not fully adhere to our selection criteria.

Third, our sample was based on a small sample of 155 OP, corresponding to 53.8% of the minimum sample size estimated a priori and approximately 7.0% of participants from the targeted discussion group. Consequently, the study should be regarded as exploratory rather than confirmatory, and the limited sample size inevitably reduced statistical power and the precision of some effect estimates, as reflected by the wide confidence intervals observed for several variables. Moreover, as the number of active users at the time of the survey was unavailable, calculation of the response rate should be cautiously appraised. Another important limitation concerns the regional distribution of participants. Our sample cannot be considered fully representative of Italian occupational physicians, particularly in a country characterized by substantial regional differences in healthcare organization and by school-specific approaches to Occupational Medicine residency training [41,78], Nevertheless, the survey ultimately included approximately 1.8% of all officially registered Italian OP (n = 8715 by 20 June 2026), suggesting that our results may still retain some significance for exploratory aims. Accordingly, the present study should be regarded as a preliminary assessment of this topic, representing an opportunity for selecting potential participants, assessing their enrolment, recording the required data, questionnaire delivery and collection, supervision and quality control, and data processing, otherwise stressing the need for future iterations and researches, and highlighting the need for larger, nationally representative studies to confirm the present findings [79].

Fourth, we cannot rule out a significant social desirability bias, particularly when dealing with knowledge test. Social desirability bias is a quite common in KAP studies and can be defined as a systematic response bias whereby participants tend to overreport socially desirable attitudes and practices, and underreport behaviors or opinions perceived as undesirable, leading to a potential overestimation of favorable knowledge, attitudes, or behaviors [24,33]. Therefore, we cannot rule out that also our results could have ultimately overstated the share of individuals having an effective understanding of endometriosis, as well as the proportion of respondents actually having issued any conditioned fitness to work appraisal.

Despite these limitations, our findings provide one of the first systematic assessments of occupational physicians’ knowledge, attitudes, and practices regarding endometriosis. Future research should focus on larger and more representative samples, evaluate the effectiveness of educational interventions, and support the development of evidence-based occupational recommendations aimed at improving workplace inclusion and health outcomes among women affected by endometriosis.

## 5. Conclusions

Italian OPs generally perceived endometriosis as a clinically relevant condition and reported substantial previous experience in managing affected workers. However, despite a relatively high degree of self-perceived familiarity, important gaps emerged regarding epidemiology, diagnostic pathways, and awareness of occupational recommendations. The issuance of conditioned fitness-for-work appraisals appeared to be influenced by factors associated with continuous professional exposure to the topic, including continuing medical education, exchanges with colleagues, and practice in healthcare settings, suggesting that undergraduate medical training alone may be insufficient to support evidence-informed occupational management. Taken together, these findings support the need for targeted educational initiatives and the development of shared recommendations aimed at improving the occupational management of women affected by endometriosis and promoting their full participation in working life.

## Figures and Tables

**Table 1:** General characteristics of the sample (Note: 95% CI = 95% confidence interval).

Variable	No./155, %	Average, 95% CI
Age (years)		43.75 (42.27; 45.24)
Seniority (years)		17.05 (15.45; 18.64)
Male Gender	91, 58.71%	
Region of origin		
Northern Italy	103, 66.45%	
Central Italy	20, 12.90%	
Southern Italy	32, 20.65%	
Qualification as Occupational physician		
Residency in Occupational Medicine	136, 87.74%	
Residency in Hygiene and Public Health	18, 11.61%	
Residency in Legal medicine	1, 0.65%	
Occupational physicians of healthcare providers	104, 67.10%	
Free practitioner	116, 74.84%	
Self-assessed proper knowledge of endometriosis	121, 78.06%	
Endometriosis acknowledged as somehow severe	110, 70.97%	
Endometriosis acknowledged as somehow frequent	86, 55.48%	
Risk Perception Score (%)		52.52 (50.64; 54.40)
General Knowledge Score (%)		64.14 (62.21; 66.07)
General Knowledge Score (simplified) (%)		60.00 (55.25; 62.17)
Previous formation on endometriosis		
During medical school	105, 67.74%	
During medical specialty training	4, 2.58%	
Continuous Medical Education	29, 18.71%	
Colleagues (Gynecologists)	47, 30.32%	
Internet (i.e., online courses, articles, official websites)	49, 31.61%	
Knowledge of any guidelines on occupational management of endometriosis	17, 10.97%	
Background in …		
Gynecology	19, 12.26%	
Psychiatry	13, 8.39%	
Internal medicine	102, 65.81%	
Any previous experience with patients with endometriosis	126, 81.29%	
Conditioned Fitness to work assessment		
Any restriction	102, 65.81%	
Avoid night shift	47, 30.32%	
Avoid shift work	36, 23.23%	
Avoid exposure to extreme temperatures	15, 9.68%	
Increased number of work breaks	78, 50.32%	
Avoid exposure to chemical agents	3, 1.94%	
Avoid front office duties	18, 11.61%	

**Table 2:** Detailed list of items included in the knowledge test and correlation of the proportion of correct answers with the cumulative general knowledge test (GKS).

Question/Statement	Correct Answer	No., %	Correlation with GKS (rho, *p* Value)
W01. What is the prevalence of endometriosis among women of reproductive age?	10 to 14%	41, 26.45%	−0.073 (0.363)
W02. Endometriosis is caused by the presence of endometrium-like tissue outside the uterus.	TRUE	155, 100%	-
W03. Endometriosis is associated with abnormalities of the pelvic musculature.	TRUE	111, 71.61%	0.172 (0.033)
W04. Early menarche is a risk factor for endometriosis.	TRUE	70, 45.16%	0.338 (<0.001)
W05. Women with endometriosis typically have a low body mass index (<18 kg/m^2^).	FALSE	86, 55.48%	0.122 (0.132)
W06. Endometriosis is characterized by a strong familial predisposition.	TRUE	54, 34.84%	0.063 (0.440)
W07. Endometriosis is usually diagnosed …	… between 25 and 35 years of age	96, 61.94%	−0.159 (0.048)
W08. The gold standard for the diagnosis of endometriosis is transvaginal ultrasound.	FALSE	49, 31.61%	0.166 (0.040)
W09. Endometriosis is frequently associated with infertility.	TRUE	129, 83.23%	0.312 (<0.001)
W10. Which of the following psychiatric disorders is most commonly associated with endometriosis?	Depression	141, 90.97%	0.128 (0.113)
W11. The treatment of endometriosis aims to reduce pain and maximise residual reproductive potential.	TRUE	127, 81.94%	−0.074 (0.362)
W12. The treatment of endometriosis benefits from hormone replacement therapy.	TRUE	120, 77.42%	−0.127 (0.114)

**Table 3:** Distribution of main variables assessed within the survey with the outcome variable of having formulated medical restrictions based on the underlying status of endometriosis (Note: 95% CI = 95% confidence interval; continuous variables were assessed by means of Mann-Whitney U test, while categorical ones were assessed by means of chi squared test).

Variable	Previously Issuing Restricted Fitness-to-Work Appraisal of Women Affected by Endometriosis		*p* Value
	Yes (N = 102)	No (N = 53)	
Gender			<0.001
Male	76, 74.51%	15, 28.30%	
Female	26, 25.49%	38, 71.70%	
Age (Average, 95% CI)	44.88 (42.97; 46.80)	41.58 (39.33; 43.84)	0.036
Seniority (Average, 95% CI)	18.37 (16.35; 20.39)	14.49 (11.99; 16.99)	0.022
Region of Italy			0.024
Northern Italy	61, 59.80%	42, 79.25%	
Central/Southern Italy	41, 40.20%	11, 20.75%	
Self assessed good knowledge	80, 78.43%	41, 77.36%	1.000
RPS > median	48, 47.06%	18, 33.96%	0.164
Simplified GKS > median	42, 41.18%	8, 15.09%	0.002
Source of information			
Medical school	55, 53.92%	50, 94.34%	<0.001
Continous Medical Education	28, 27.45%	1, 1.89%	<0.001
Medical Specialty Training	3, 2.94%	1, 1.89%	1.000
Colleagues	41, 40.20%	6, 11.32%	<0.001
Internet, social media	39, 38.24%	10, 18.87%	0.023
Any background in …			
… gynecology	14, 13.73%	5, 9.43%	0.607
… psychiatry	7, 6.86%	6, 11.32%	0.519
… internal medicine	68, 66.67%	34, 64.15%	0.893
Occupational features of respondents			
Full residency in Occupational Medicine	89, 87.25%	47, 88.68%	1.000
Being a free practitioner	78, 76.47%	38, 71.70%	0.650
Working as Occupational Physician of Healthcare provider(s)	85, 83.33%	19, 35.85%	<0.001

**Table 4:** Distribution of attitudes and perceptions towards endometriosis and its management with the outcome variable of having formulated medical restrictions based on the underlying status of endometriosis (Note: 95% CI = 95% confidence interval; continuous variables were assessed by means of Mann-Whitney U test, while categorical ones were assessed by means of chi squared test).

Variable	Previously Issuing Restricted Fitness-to-Work Appraisal of Women Affected by Endometriosis		*p* Value
	Yes (N = 102)	No (N = 53)	
Perceived difficulty in the managing of endometriosis in the workplaces	6.33 (5.98; 6.68)	5.98 (5.52; 6.44)	0.105
Clinical features considered often/always associated with endometriosis			
“Intense dysmenorrhea”	94, 92.16%	52, 98.11%	0.253
“Severe dyspareunemia”	66, 64.71%	43, 81.13%	0.053
“Catamenial painful defecation”	96, 94.12%	50, 94.34%	1.000
“Chronic pelvic pain”	95, 93.14%	50, 94.34%	1.000
“Acute pelvic pain”	102, 100%	53, 100%	-
“Catamenial digestive disorders”	73, 71.57%	27, 50.94%	0.018
“Catamenial urinary function disorders”	78, 76.47%	38, 71.70%	0.650
“Menstrual cycle disorders”	95, 93.14%	49, 92.45%	1.000
“Fertility disorders”	97, 95.10%	47, 88.68%	0.252
Main barriers to workplace management of patients affected by endometriosis (complex/very complex vs. simple to moderately complex)			
Ergonomics of the workstation	30, 29.41%	7, 13.21%	0.041
Working hours	40, 39.22%	5, 9.43%	<0.001
Workload	77, 75.49%	11, 20.75%	<0.001
Work environment	23, 22.55%	11, 20.75%	0.959
Psychosocial factors	54, 52.94%	40, 75.47%	0.011
Interface between family and workplace	74, 72.55%	10, 18.87%	<0.001
Other occupational risk factors	31, 30.39%	3, 5.66%	<0.001

**Table 5:** Hierarchical multivariable logistic regression analysis according to the Knowledge–Attitudes–Practices (KAP) framework for factors associated with previous issuance of conditioned fitness-to-work appraisals. Explanatory variables were hierarchically included as follows: demographic characteristics (Model I), knowledge-related variables (Model II), attitudes (Model III), and professional characteristics (Model IV) (note: OR = odds ratio; 95% CI = 95% confidence interval; RPS = risk perception score; sGKS = simplified general knowledge score; OP = occupational physician).

Variable	MODEL I (OR, 95% CI)	MODEL II (OR, 95% CI)	MODEL III (OR, 95% CI)	MODEL IV (OR, 95% CI)
Age (years)	1.032 (0.987; 1.078)	1.066 (0.992; 1.146)	1.071 (0.994; 1.153)	1.048 (0.966; 1.136)
Seniority (years)	1.026 (0.983; 1.070)	0.975 (0.916; 1.038)	0.974 (0.915; 1.038)	0.962 (0.896; 1.032)
Male Gender	6.420 (2.986; 13.803)	7.091 (2.202; 22.829)	7.172 (2.221; 23.168)	5.584 (1.576; 19.789)
Being from Northern Italy	2.119 (0.896; 5.010)	1.211 (0.320; 4.588)	1.201 (0.319; 4.521)	1.048 (0.259; 4.232)
RPS > median value	-	-	0.699 (0.199; 2.456)	0.876 (0.212; 3.624)
sGKS > median value	-	0.524 (0.139; 1.972)	0.556 (0.146; 2.127)	0.590 (0.135; 2.588)
Having managed any case of endometriosis	-	-	-	0.897 (0.152; 5.305)
Any background in gynecology	-	0.547 (0.099; 3.018)	0.551 (0.101; 3.003)	1.071 (0.143; 8.039)
Any background in psychiatry	-	1.197 (0.131; 10.916)	1.325 (0.139; 12.653)	2.667 (0.212; 33.516)
Any background in internal medicine	-	0.450 (0.118; 1.708)	0.440 (0.116; 1.668)	0.634 (0.135; 2.972)
Full residency in Occupational Medicine	-	-	-	0.716 (0.129; 3.974)
Being a free practitioner	-	-	-	0.382 (0.094; 1.550)
Working as OP for healthcare providers	-	-	-	9.546 (2.292; 39.761)
Source of information: medical school	-	0.050 (0.009; 0.271)	0.054 (0.010; 0.297)	0.048 (0.007; 0.322)
Source of information: CME	-	125.674 (10.030; 1574.602)	142.295 (10.730; 1887.010)	176.119 (9.487; 3269.523)
Source of information: medical specialty training	-	55.746 (2.264; 1372.890)	45.939 (1.711; 1233.352)	126.171 (2.470; 6445.337)
Source of information: colleagues	-	5.607 (1.431; 21.969)	5.186 (1.292; 20.814)	7.143 (1.316; 38.782)
Source of information: internet	-	3.746 (1.061; 13.220)	4.015 (1.101; 14.644)	3.820 (0.867; 16.820)

**Figure A3:**
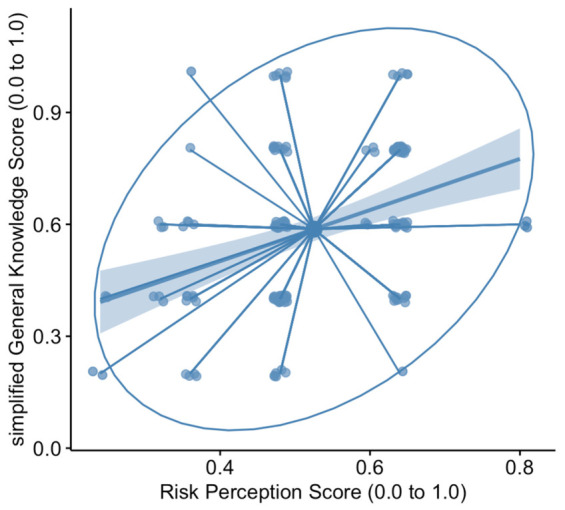
Correlation between the Risk Perception Score and the General Knowledge Score (rho = 0.372, 95% confidence interval 0.230 to 0.501; *p* < 0.001). The ellipse represents the 95% confidence ellipse derived from the covariance matrix of the two variables and provides a graphical summary of the joint distribution of the observations.

## Data Availability

Raw data are available upon request to the principal investigator.

## Supplementary Materials

The following supporting information can be downloaded at: https://www.mdpi.com/article/10.3390/healthcare14152299/s1, File S1: STROBE Checklist. File S2, Questionnaire translation.

## Abbreviations

The following abbreviations are used in this manuscript:

95% CI	95% Confidence Interval
aOR	Adjusted Odds Ratio
ANOVA	Analysis of Variance
CME	Continuing Medical Education
GKS	General Knowledge Score (sGKS, simplified GKS)
HBM	Health Belief Model
KAP	Knowledge, Attitudes and Practices
OP	Occupational Physician
OR	Odds Ratio
RPS	Risk Perception Score
STROBE	Strengthening the Reporting of Observational Studies in Epidemiology

## Appendix A

**Table A1:** Distribution of reported clinical features of endometriosis and perceived barriers towards the management of endometriosis on the workplaces by having or not continuous medical education (CME) activities as the main source of information on endometriosis, having or not a full residency in Occupational Medicine, working or not as occupational physician (OP)s for any healthcare provider. All comparisons were performed by means of chi squared test.

Variable	CME as Main Source of Information on Endometriosis		*p* Value	Full Residency in Occupational Medicine		*p* Value	Working as OPs for Healthcare Provider(s)		*p* Value
	Yes (N = 29)	No (N = 126)		Yes (N = 136)	No (N = 19)		Yes (N = 44)	No (N = 111)	
Clinical features considered as often/always associated with endometriosis									
“Intense dysmenorrhea”	26, 89.66%	120, 95.24%	0.472	130, 95.59%	16, 84.21%	0.144	41, 93.18%	105, 94.59%	1.000
“Severe dyspareunemia”	18, 62.07%	91, 72.22%	0.393	97, 71.32%	12, 63.16%	0.644	32, 72.73%	77, 69.37%	0.828
“Catamenial painful defecation”	20, 68.97%	80, 63.49%	0.734	92, 67.65%	8, 42.11%	0.054	26, 59.09%	74, 66.67%	0.482
“Chronic pelvic pain”	27, 93.10%	118, 93.65%	1.000	129, 94.85%	16, 84.21%	0.204	41, 93.18%	104, 93.69%	1.000
“Acute pelvic pain”	102, 100%	53, 100%	-	136, 100%	19, 100%	-	44, 100%	111, 100%	-
“Catamenial digestive disorders”	28, 96.55%	118, 93.65%	0.871	128, 94.12%	18, 94.74%	1.000	40, 90.91%	106, 95.50%	0.472
“Catamenial urinary function disorders”	26, 89.66%	90, 71.43%	0.072	103, 75.74%	13, 68.42%	0.685	35, 79.55%	81, 72.97%	0.591
“Menstrual cycle disorders”	26, 89.66%	118, 93.65%	0.723	128, 94.12%	16, 84.21%	0.272	44, 100%	100, 90.09%	0.069
“Fertility disorders”	29, 100%	115, 91.27%	0.211	127, 93.38%	17, 89.47%	0.885	41, 93.18%	103, 92.79%	1.000
Main barriers to workplace management of patients affected by endometriosis (complex/very complex vs. simple to moderately complex)									
Ergonomics of the workstation	9, 31.03%	28, 22.2%	0.446	33, 24.26%	4, 21.05%	0.984	12, 27.27%	25, 22.52%	0.677
Working hours	14, 48.28%	31, 24.60%	0.021	39, 28.68%	6, 31.58%	1.000	9, 20.45%	36, 32.43%	0.199
Workload	20, 68.97%	68, 53.97%	0.207	74, 54.41%	14, 73.68%	0.180	25, 56.82%	63, 56.76%	1.000
Work environment	4, 13.79%	30, 23.81%	0.354	30, 22.06%	4, 21.05%	1.000	8, 18.18%	26, 23.42%	0.620
Psychosocial factors	10, 34.48%	84, 66.67%	0.003	88, 64.71%	6, 31.58%	0.012	25, 56.82%	69, 62.16%	0.666
Interface between family and workplace	17, 58.62%	67, 53.17%	0.746	74, 54.41%	10, 52.63%	1.000	26, 59.09%	58, 52.25%	0.554
Other occupational risk factors	10, 34.48%	24, 19.05%	0.118	31, 22.79%	3, 15.79%	0.693	9, 20.45%	25, 22.52%	0.948

**Table A2:** Summary of main analysis, performed by means of binary logistic regression, and sensitivity analysis, including two distinctive models, i.e., a general linear model and a Firth logistic regression model. In main analysis, only explanatory variables being associated with outcome variable for a *p* value < 0.05 in univariate analysis were included. In both sensitivity analysis models, all variables were included irrespective of results from univariate analysis. All results are reported as adjusted odds ratios with their respective 95% confidence intervals.

Variable	Main Analysis (Binary Logistic Regressio)	Sensitivity Analsysis (GLM)	Sensitivity Analsysis (Firth Logistic Regression)
Age (years)	1.040 (0.971; 1.114)	1.039 (0.961; 1.129)	1.029 (0.962; 1.105)
Seniority (years)	0.981 (0.923; 1.043)	0.959 (0.889; 1.027)	0.974 (0.916; 1.032)
Male Gender	5.685 (1.839; 17.579)	6.002 (1.766; 24.088)	3.843 (1.356; 12.125)
Being from Northern Italy	1.079 (0.299; 3.891)	0.922 (0.222; 3.924)	0.908 (0.265; 3.185)
RPS > median value	-	1.08 (0.246; 4.517)	1.076 (0.304; 3.623)
sGKS > median value	1.449 (0.385; 5.463)	1.611 (0.347; 7.226)	1.476 (0.393; 5.321)
Self assessed good knowledge	-	3.882 (0.745; 24.271)	2.562 (0.632; 11.808)
Any background in gynecology	-	0.709 (0.085; 6.078)	0.759 (0.127; 4.723)
Any background in psychiatry	-	0.303 (0.023; 4.119)	0.402 (0.047; 3.704)
Any background in internal medicine	-	1.785 (0.368; 9.081)	1.509 (0.387; 6.056)
Full residency in Occupational Medicine	-	0.854 (0.126; 5.490)	0.901 (0.173; 4.414)
Being a free practitioner	-	2.658 (0.671; 11.599)	2.05 (0.626; 7.183)
Working in Healthcare settings	5.909 (1.923; 18.162)	14.541 (3.415; 83.191)	7.392 (2.220; 29.941)
Source of information: medical school	0.073 (0.014; 0.388)	0.035 (0.004; 0.201)	0.087 (0.015; 0.356)
Source of information: CME	85.720 (6.595; 1114.208)	170.831 (15.462; 5597.089)	44.31 (6.651; 607.977)
Source of information: medical specialty training	-	278.444 (5.097; 56,366.359)	61.234 (2.325; 4756.903)
Source of information: colleagues	4.669 (1.226; 17.774)	8.242 (1.581; 58.569)	4.895 (1.232; 24.501)
Source of information: internet	2.714 (0.767; 9.599)	3.472 (0.78; 15.945)	2.609 (0.735; 9.261)
