# Supplementary material for: Management of Endometriosis in the Workplaces: Preliminary Results from a Cross-Sectional Knowledge, Attitudes and Practices Study (Italy, 2026)
Source: Healthcare (Basel). 2026 Jul 29;14(15):2299. doi: 10.3390/healthcare14152299 (PMC13466194; doi:10.3390/healthcare14152299)
Supplement: Supplementary file 1 [file healthcare-14-02299-s001.zip › Supplementary File S1 - STROBE Checklist.pdf]

STROBE Statement—Checklist of items that should be included in reports of cross-sectional studies

Item	Recommendation	Reported in manuscript
**Title and Abstract**		
1a	Indicate the study's design with a commonly used term in the title or the abstract	Yes (cross-sectional survey reported in Abstract and Methods 2.1)
1b	Provide in the abstract an informative and balanced summary	Yes
**Introduction**		
2	Explain the scientific background and rationale	Introduction
3	State specific objectives and prespecified hypotheses	Last paragraph of Introduction
**Methods**		
4	Present key elements of study design early in the paper	Section 2.1
5	Describe the setting, locations, and relevant dates	Section 2.1
6a	Give eligibility criteria and sources/methods of selection of participants	Section 2.1
7	Clearly define outcomes, exposures, predictors, confounders	Sections 2.3–2.5
8*	Data sources/measurement methods	Section 2.3
9	Describe efforts to address potential bias	Sections 2.1, 2.3, 2.4, Discussion-Limitations
10	Explain how study size was determined	Section 2.2
11	Explain handling of quantitative variables	Section 2.5
12a	Describe all statistical methods	Section 2.5
12b	Methods used to examine	Logistic regression, Section 2.5

Item	Recommendation	Reported in manuscript
	subgroups/interactions	
12c	Explain handling of missing data	Not applicable (mandatory responses in Google Forms)
12d	Analytical methods accounting for sampling strategy	Not applicable
12e	Sensitivity analyses	Not performed
**Results**		
13a	Report numbers of individuals at each stage	Section 3.1
13b	Give reasons for non-participation	Section 3.1
13c	Consider use of flow diagram	Appendix Figure A1
14a	Give characteristics of study participants	Table 1
14b	Indicate number with missing data	No missing data
15	Report outcome events or summary measures	Tables 2–5
16a	Give unadjusted and adjusted estimates with precision	Tables 4–5
16b	Report category boundaries if continuous variables categorized	Section 2.5
16c	Consider translating estimates into absolute risk	Not applicable
17	Report other analyses done	Cronbach's alpha; Spearman correlation; logistic regression
**Discussion**		
18	Summarize key results with reference to objectives	Discussion, Section 4.1
19	Discuss limitations, bias and imprecision	Discussion, Limitations
20	Provide cautious overall interpretation	Discussion
21	Discuss generalizability	Discussion
**Other information**		

Item	Recommendation	Reported in manuscript
22	Give source of funding and role of funders	No external funding
23	Ethics approval and informed consent	Section 2.4

*Information from separately reported studies should be cited where appropriate.
