# Supplementary material for: Management of Endometriosis in the Workplaces: Preliminary Results from a Cross-Sectional Knowledge, Attitudes and Practices Study (Italy, 2026)
Source: Healthcare (Basel). 2026 Jul 29;14(15):2299. doi: 10.3390/healthcare14152299 (PMC13466194; doi:10.3390/healthcare14152299)
Supplement: Supplementary file 1 [file healthcare-14-02299-s001.zip › Supplementary File S2 - Questionnaire translation.pdf]

ENDOMETRIOSIS AND WORK

Your experience with endometriosis in the workplace

Dear colleague,

Information notice

This anonymous questionnaire aims to investigate your knowledge regarding the management of endometriosis in the field of occupational medicine. Participation is entirely voluntary. The information requested is collected in generic form only, and the data obtained from the analysis will be processed anonymously, without any possibility of tracing responses back to the respondent (no e-mail addresses or IP addresses from computers/mobile phones/tablets used to complete the questionnaire will be collected), in full compliance with current privacy regulations.

The data collected are entirely generic, necessary only for demographic analyses (sex, age, etc.), and cannot in any way be linked to the identity of the respondent.

In accordance with European Regulation 2016/679 (GDPR), we further inform you that:

- The data controller and person responsible for data protection before, during, and after the analysis process is Dr. Matteo Riccò, who may be contacted at: mricco2000@gmail.com;
- Data collected will be used exclusively for the scientific purposes described above;
- Unless specifically required by judicial authorities in accordance with applicable laws, data will not be shared with third parties or disseminated outside the scientific research process;
- Once the questionnaire has been completed, it will not be possible to identify the respondent. As the questionnaire is fully anonymized, modifications, corrections, or deletion of submitted responses cannot be requested;
- Data will be retained only for the time strictly necessary to complete the study.

Do you agree to participate in this study?

○ Yes
○ No

Q10A

Do you believe that you have adequate knowledge of endometriosis?

○ Yes
○ No
○ I do not remember

Q10B

How did you acquire your knowledge about endometriosis?
(Multiple answers allowed)

□ Undergraduate medical education
□ Continuing Medical Education (CME)
□ Residency training

□ Gynecologist colleagues
□ Internet resources

Q10C

To your knowledge, are there any guidelines addressing the management of endometriosis in the workplace?

○ Yes
○ No
○ I do not remember

Q11

In your opinion, endometriosis is a condition that is:

1 = Very rare
5 = Very common

Q12

In your opinion, endometriosis is a condition that is:

1 = Very mild
5 = Very severe

Management of endometriosis cases in the workplace

Q13

Have you ever managed workers affected by endometriosis?

○ Yes
○ No
○ Prefer not to answer

Q13A

How many workers affected by endometriosis have you managed in the past year?

○ Fewer than 5
○ 5–10
○ More than 10
○ None

Q14

The workers were clinically followed for endometriosis by:

(Multiple answers allowed)

□ General practitioner
□ Outpatient specialists
□ Other
□ None
□ Not applicable

Q15

Have you ever requested diagnostic investigations that led to a diagnosis of endometriosis in a worker who had not previously been diagnosed?

○ Yes
○ No
○ Prefer not to answer

Q16

Have you ever requested specific diagnostic investigations or specialist consultations for workers with a known diagnosis of endometriosis?

○ Yes
○ No
○ Prefer not to answer
○ Not applicable (I have never managed cases of endometriosis)

Q17

Have you ever established a specific health surveillance protocol for workers affected by endometriosis?

○ Yes
○ No
○ Prefer not to answer

Q18

Have you ever prescribed specific restrictions or accommodations for workers affected by endometriosis?

○ Yes
○ No
○ Prefer not to answer

Q19

If yes, for which domains/risk factors?

(Multiple answers allowed)

□ Night work
□ Shift work
□ Exposure to high or low temperatures
□ Exposure to intense light
□ Exposure to dust
□ Front-office/customer-facing activities
□ Work breaks
□ Exposure to chemical agents

Q20A

Have you ever received requests for extraordinary medical examinations from workers affected by endometriosis?

○ No
○ Yes, at least one
○ Yes, more than one
○ Prefer not to answer

Q21

Have you ever issued an unfit-for-work judgment due to endometriosis?

○ No
○ Yes, at least one
○ Yes, more than one
○ Prefer not to answer

Q22

Have you ever received appeals against your fitness-for-work judgments (Art. 41, paragraph 9) concerning endometriosis?

○ No
○ Yes, at least one
○ Yes, more than one
○ Prefer not to answer

In your experience, the most difficult issues to manage in workers affected by endometriosis are:

(1 = Totally disagree; 5 = Totally agree)

Q23A Ergonomics of the workstation

Q23B Mitigation of occupational risk factors

Q23C Working hours

Q23D Work pace

Q23E Characteristics of the workplace

Q23F Work-related stress / psychosocial factors

Q23G Work–life interface and interaction with individual risk factors

Knowledge Test

To your knowledge...

W01. What proportion of women of reproductive age is affected by endometriosis?

○ <5%
○ 5–9%
○ 10–14%
○ 15–20%
○ >20%
○ I do not know

W02. Endometriosis is caused by the presence of endometrial-like tissue outside the uterus.

○ True
○ False
○ I do not know

W03. Endometriosis is associated with abnormalities of the pelvic musculature.

○ True
○ False
○ I do not know

W04. Early menarche is a risk factor for endometriosis.

○ True
○ False
○ I do not know

W05. Women affected by endometriosis usually have a low body mass index (<18 kg/m²).

○ True
○ False
○ I do not know

W06. Endometriosis is characterized by a strong familial predisposition.

○ True
○ False
○ I do not know

W07. Endometriosis is usually diagnosed...

○ Between 12 and 14 years of age
○ Between 15 and 21 years of age
○ Between 25 and 35 years of age
○ Between 35 and 45 years of age
○ I do not know

W08. The gold standard for the diagnosis of endometriosis is transvaginal ultrasound.

○ True
○ False
○ I do not know

W09. Endometriosis is frequently associated with infertility.

○ True
○ False
○ I do not know

W10. Which of the following psychiatric disorders is most frequently associated with endometriosis?

○ Bipolar disorder
○ Depression
○ Paranoid personality disorder
○ Obsessive-compulsive disorder

W11. The treatment of endometriosis aims to reduce pain and maximize residual reproductive capacity.

○ True
○ False
○ I do not know

W12. Endometriosis benefits from treatment with hormone replacement therapy.

○ True
○ False
○ I do not know

Which of the following signs and symptoms are associated with endometriosis?

Response options:

- Never
- Sometimes
- Often
- Always
- I do not know

W13. Severe dysmenorrhea

W14. Acute pelvic pain

W15. Chronic pelvic pain

W16. Catamenial digestive disorders

W17. Catamenial abdominal pain

W18. Catamenial urinary symptoms

W19. Menstrual cycle disorders

W20. Fertility disorders

W21. Severe dyspareunia

In your experience as an occupational physician, what degree of complexity do you attribute to the management of the following conditions when issuing fitness-for-work judgments?

Scale:

1 = Very low complexity

10 = Very high complexity

D01. Endometriosis

D02. Diabetes mellitus

D03. Allergic asthma

D04. Chronic low back pain / Lumbar disc herniation

D05. Upper limb disorders

D06. Ischemic heart disease

D07. Fibromyalgia

D08. Depression

D09. Epilepsy

D10. Migraine

Finally, a few questions about yourself...

Q01. Year of birth

Q02. Year of graduation

Q03. You are:

○ Male

○ Female

○ Prefer not to answer

Q04. In which geographical area do you mainly work as an occupational physician?

○ Northern Italy (Aosta Valley, Piedmont, Liguria, Lombardy, Veneto, Autonomous Province of Trento, Autonomous Province of Bolzano, Friuli Venezia Giulia, Emilia-Romagna)

○ Central Italy (Tuscany, Umbria, Marche, Abruzzo, Lazio)

○ Southern Italy / Islands (Campania, Molise, Apulia, Basilicata, Calabria, Sicily, Sardinia)

○ Prefer not to answer

Q06. During your training, did you receive at least six months of training in:

(Multiple answers allowed)

□ Gynecology department/outpatient clinic

□ Psychiatry department/outpatient clinic

□ Internal medicine department

Q07. You are qualified as:

(Multiple answers allowed)

□ Specialist in Occupational Medicine

□ Specialist in Hygiene and Preventive Medicine

□ Specialist in Legal Medicine

□ Qualified pursuant to Italian Law 277/91, Article 55

Q08. You currently work as an occupational physician as:

○ Self-employed professional

○ Employee / external consultant

○ Both self-employed professional and external consultant

Q09. As an occupational physician, do you provide services to healthcare facilities?

○ No

○ Yes, private facilities only

○ Yes, public facilities only

○ Yes, both public and private facilities

○ Prefer not to answer
